# Delineation of the Ancestral Tus-Dependent Replication Fork Trap

**DOI:** 10.3390/ijms222413533

**Published:** 2021-12-16

**Authors:** Casey J. Toft, Morgane J. J. Moreau, Jiri Perutka, Savitri Mandapati, Peter Enyeart, Alanna E. Sorenson, Andrew D. Ellington, Patrick M. Schaeffer

**Affiliations:** 1 Molecular and Cell Biology, College of Public Health, Medical and Veterinary Sciences, James Cook University, Douglas, QLD 4811, Australia; casey.toft@jcu.edu.au (C.J.T.); morgane.moreau@gmail.com (M.J.J.M.); alanna.sorenson@jcu.edu.au (A.E.S.); 2 Centre of Tropical Bioinformatics and Molecular Biology, James Cook University, Douglas, QLD 4811, Australia; 3 Institute for Cell and Molecular Biology, University of Texas, Austin, TX 78712, USA; jperutka@gmail.com (J.P.); savitrimandapati@gmail.com (S.M.); enyeartpj@gmail.com (P.E.); ellingtonlab@gmail.com (A.D.E.)

**Keywords:** replication fork trap, Tus–*Ter*, *dif*, ChIP-Seq, GC-skew, Enterobacterales

## Abstract

In *Escherichia coli*, DNA replication termination is orchestrated by two clusters of *Ter* sites forming a DNA replication fork trap when bound by Tus proteins. The formation of a ‘locked’ Tus–*Ter* complex is essential for halting incoming DNA replication forks. However, the absence of replication fork arrest at some *Ter* sites raised questions about their significance. In this study, we examined the genome-wide distribution of Tus and found that only the six innermost *Ter* sites (*TerA–E* and *G*) were significantly bound by Tus. We also found that a single ectopic insertion of *TerB* in its non-permissive orientation could not be achieved, advocating against a need for ‘back-up’ *Ter* sites. Finally, examination of the genomes of a variety of Enterobacterales revealed a new replication fork trap architecture mostly found outside the Enterobacteriaceae family. Taken together, our data enabled the delineation of a narrow ancestral Tus-dependent DNA replication fork trap consisting of only two *Ter* sites.

## 1. Introduction

**Figure 1:**
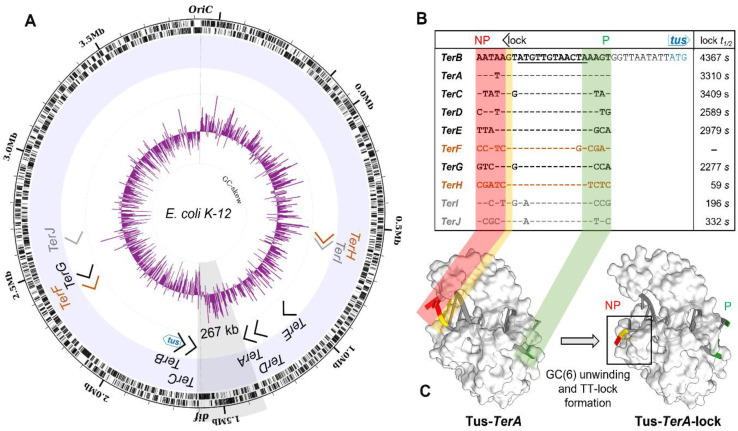
Chromosomal distribution and sequences of *Ter* sites in *E. coli*. (**A**) Circular representation of *E. coli* K12 MG1655. Illustrated from the outside to the centre of the circle: labelled forward and reverse genes; location of the ten primary *Ter*-sites (*TerA*–*J*) and their strand orientation; the currently accepted replication termination fork trap involving high-affinity (black), moderate-affinity (grey) and non-lock forming *Ter* sites (orange); GC-skew (purple) over a 5000 bp window showing a switch in polarity at the replication origin (*oriC*) and close to *TerC* and the *dif* site. (**B**) *Ter* site sequences with the G(6) base complementary to C(6) highlighted in yellow and the strictly conserved 12 bp core sequence (underlined). *TerB* is located 11 bp upstream of the start codon (ATG) of the *tus* gene. Half-lifes (*t_1_*_/*2*_) of Tus–*Ter*–lock complexes at 250 mM KCl were described previously [5]. (**C**) DNA replication fork arrest at a *Ter* site. The unwinding action of DnaB helicase breaks the GC(6) base pair at the non-permissive (NP: red) end of a Tus-bound *Ter*. The C(6) docks into the cytosine-specific binding pocket (boxed) forming an extremely stable Tus–*Ter*–lock complex. Permissive (P) end of *Ter*: green.

Bacteria such as *Escherichia coli* and *Bacillus subtilis* utilise distinct DNA replication fork trap systems within their chromosomal terminus region [1]. This is exemplified in *E. coli* by the presence of a cluster of five similar but distinct 23-bp *Ter* DNA sequences on each chromosomal arm, which have anti-helicase activity when they are bound by the replication termination protein Tus [1,2,3]. The complexity of the *E. coli* replication fork trap with respect to multiplicity and wide distribution of *Ter* sites around the chromosome is puzzling. One cluster consisting of *TerB*, *C*, *F*, *G* and *J* arrests the clockwise moving replication fork. The second cluster, which is oppositely oriented, consists of *TerA*, *D*, *E*, *I*, *H* and arrests the anticlockwise moving replication fork (Figure 1A). Until recently, the notions that Tus could bind to all ten of these slightly different *Ter* DNA sequences (*TerA–J*) (Figure 1B), and that these sequences all have a significant role in replication termination, have remained mostly unchallenged despite their individual binding properties for Tus being significantly different. Each *Ter* cluster consists of three high-affinity, one moderate-to-low-affinity and one non-lock forming *Ter* site (Figure 1A) [4,5]. Four additional *Ter*-like sequences (*TerK*, *L*, *Y* and *Z*) can be found in the *E. coli* chromosome, one within the previously identified termination region and the other three being on the left part of the chromosome, but these were dismissed as pseudo-*Ter* sites [6]. Binding of Tus to the pseudo-*Ter* sites is likely to be insignificant based on their sequences [5] and fork arrest efficiency [6].

The unique mechanism of polar DNA replication fork arrest observed in *E. coli* is due to the unusual binding mode of Tus to *Ter* and the unwinding action of the DnaB helicase at the non-permissive face of the Tus–*Ter* complex (Figure 1C) [1,6,7,8,9,10,11]. Although a specific protein–protein interaction between the DnaB helicase and Tus had initially been proposed to have a pivotal role in polar fork arrest [7,12], several studies have shown that this interaction is not necessary for polar fork arrest at the non-permissive face of the Tus–*Ter* complex [5,9,13,14,15]. 

Tus precisely and tightly binds onto a *Ter* site, bending the DNA to prepare the molecular mouse trap that will be triggered by the 5’-3’ translocation and DNA unwinding action of DnaB helicase on the lagging strand moving towards the non-permissive face of the Tus–*Ter* complex [5,9,16]. The progressive separation of DNA strands at the non-permissive face of the Tus–*Ter* complex ultimately breaks the GC(6) base pair (Figure 1B) in the *Ter* core sequence, leading to the precise docking of the freed C(6) into a cytosine-specific binding pocket on the surface of Tus (Figure 1C). The formation of the locked Tus–*Ter* conformation (Tus–*Ter*–lock) slows the dissociation of Tus considerably and is believed to inhibit further DnaB helicase translocation [5,8,9].

Moreau and Schaeffer applied three different approaches to examine the kinetic and equilibrium parameters of all ten Tus–*Ter* and locked complexes in further detail (Figure 1B) [5,14,17]. They proposed a sequential three-step model for fork arrest including initial non-specific ‘sliding’ of Tus on DNA mediated by weak cooperative electrostatic interactions, followed by proper ratchet-like docking of Tus onto *Ter* upon correct alignment of specific nucleotide-amino acid contacts, and finally, the DnaB-induced Tus–*Ter*–lock via binding of C(6) to the cytosine binding-pocket of Tus [5]. The same study provided a new classification of *Ter* sites based on their kinetic and affinity parameters as well as their capacity to form a locked complex and challenged the status quo by rejecting *TerF* and *TerH* as functional *Ter* sequences, arguing that they could not induce polar fork arrest due to their inability to form a locked complex. A substitution of the canonical T to G at position 5 in the core sequence (Figure 1B) was proposed to be the cause for this loss of function [5]. In addition, Coskun-Ari and Hill had previously observed a significant loss of replication fork arrest activity when *TerB* was mutated at TA(9) to CG or GC [18]. *TerI* and *TerJ* are the only *Ter* sites where the TA(9) is replaced by an AT, which, taken together with the relatively fast Tus dissociation from these locked Tus–*Ter* complexes [5], also raises doubts about their actual role in replication termination under natural Tus abundance conditions.

Using two-dimensional gel analysis of replication intermediates at *Ter* sites under natural conditions, Duggin and Bell showed that *TerC* is the most frequently used, with significant fork pausing also observed at *TerA* and *TerB* [6]. Tus over-expression was required to observe intermediates at some of the other *Ter* sites [6]. The sites varied significantly in their capacity to arrest replication forks, and this was later correlated to their respective affinity for Tus (high, moderate or weak) and their ability to form a Tus–*Ter*–lock [4,5,17]. Most puzzling, however, was the observation that some pausing occurred at the outer *TerH.* Indeed, to be arrested at *TerH*, the replication fork has to break through the strong *TerE* and moderate *TerI* but no fork pausing was observed at *TerE* and little at *TerI*. Duggin and Bell also showed that pausing was abolished at *TerC* in a *tus* null strain, but they did not verify if the pausing observed at the outermost *TerH–I* sites was also strictly due to Tus binding [6]. The low probability of the anticlockwise fork reaching *TerH*, the absence of pausing at the strong *TerE* [6] and the non-Tus–*Ter*–lock forming characteristic of *TerH* [5], suggest that the pausing observed at *TerH* could either be due to a clockwise moving fork at the permissive face of *TerH* or to recombination events [19,20,21]. 

The presence of the distal *Ter* sites and their involvement in DNA replication termination remains unclear. Forks most frequently meet at *TerC* and, to some extent, at *TerA* and *B* [6]. Assuming the two forks progress at equivalent rates, forks are more likely to meet at *TerC* than at *TerA* since *TerC* is almost perfectly located directly opposite to *oriC*, whereas the anticlockwise moving fork must travel an additional ~259 kb to encounter the non-permissive face of the Tus–*TerA* complex. Despite the stability of the locked Tus–*TerC* complex [5] in over-expressed Tus conditions, significant pausing still occurs at *TerB* and, to some extent, at *TerG* [6]. One possible explanation for pausing at *TerB* is that in some cases, the ratchet-lock mechanism [5] fails to form and the next site serves as a back-up for DNA replication arrest. In support of this, single molecule DNA replication assays suggest that a replication fork approaching a non-permissive *TerB* will fail to be arrested 52% of the time because of an inefficient Tus–*Ter*–lock mechanism [15]. The authors proposed that lock formation is dependent on transient fork stoppage by an Arg198 interaction that buys time for C(6) to dock into its binding pocket [15]. Hence, the critical need for back-up *Ter* sites throughout the terminus of the genome is for replication forks that have breached the innermost *TerA* and *TerC* sites.

So far, the large number of binding, structural and single molecule studies designed to thoroughly examine the Tus–*Ter* complexes [2,4,5,14,15], as well as fluorescence imaging aiming at examining the progression and pausing of replication forks at natural replication barriers in live bacteria [22], have failed to provide a clear explanation for the need of such a large replication fork trap in *E. coli*. In fact, the most significant knowledge gap on Tus–*Ter* replication fork traps, i.e., the binding distribution of Tus to individual *Ter* sites in replicating bacteria, has not been addressed. As such, the function of distal *Ter* sites and their biological relevance remains unclear and calls into question as to whether or not Tus proteins [23,24] really bind these *Ter* sites, and if yes, then to what extent can they block fork progression?

First, we examined the impact of a selection of *Ter* sites (*TerB*, *H* and *J*) inserted into a safe chromosomal locus in both permissive and non-permissive orientations as well as the genome-wide distribution of Tus using ChIP-Seq and ChIP-qPCR to identify the functional *Ter* sites capable of halting replication forks in *E. coli*. The data suggest that only six *Ter* sites can bind Tus and efficiently block replication fork progression in *E. coli*. Next, we examined the fork trap architecture in closely, moderately, as well as distantly related bacteria harbouring the *tus* gene to gain insight into the differential distribution of *Ter* sites. Here, we identified a new type of replication fork trap architecture that is almost exclusively and ubiquitously found outside the Enterobacteriaceae family within Enterobacterales. Taken together, our data enabled the delineation of an ancestral Tus-dependent DNA replication fork trap consisting of only two *Ter* sites, immediately suggesting a possible route to the more complex replication fork traps observed in Enterobacteriaceae. 

## 2. Results

### 2.1. Ectopic Insertion of TerB, TerH and TerJ Sites

The role of some of the distal *Ter* sites in replication fork arrest is questionable in light of their location, Tus binding affinity and dissociation kinetics [5]. *TerF* has recently been dismissed as a pseudo-*Ter* site with no possible role in replication fork arrest. While *TerH* cannot form a locked complex, the locked Tus–*TerJ* complex has a dissociation half-life (t_1/2_) of 332 s that matches the non-locked Tus–*TerB* t_1/2_ = 315 s at 250 mM KCl [5]. These findings prompted us to examine the capacity of the most distal *Ter* sites (i.e., *TerH* and *J*) to halt DNA replication forks. For this, *TerH* (moderate affinity, non-Tus–*Ter*–lock forming sequence, t_1/2_ = 59 s), *TerJ* (weakest moderate affinity, weak Tus–*Ter*–lock forming sequence, t_1/2_ = 332 s) and the strong Tus–*Ter*–lock forming *TerB* (t_1/2_ = 4367 s) were inserted in the right chromosome arm of *E. coli* strain BL21(*DE3*), 930 kbp downstream of *oriC* (right arm, SIR5.6) in both permissive or non-permissive orientations using a TargeTron strategy [25]. While ectopic *Ter* site insertions and fork trap inversions have been studied previously [26,27,28,29], the TargeTron technique guarantees that a 23 bp *Ter* within a short intron sequence is incorporated with minimal genomic variations. We hypothesised that *Ter* insertions resulting in weak to moderate replication fork pausing would yield a measurable effect on bacterial growth rate, while *Ter* insertions yielding efficient ectopic fork arrest should be unviable. As such, ectopic insertion of *TerB* should be fully viable in permissive orientation and unviable in non-permissive orientation if the Tus–*Ter*–lock is unbreachable.

Growth rates were determined for viable *E. coli* cells with successful ectopic *Ter* sites insertions confirmed by sequencing (Table 1). All *Ter* sites, except *TerB* in non-permissive orientation, could be inserted in either permissive or non-permissive orientation into SIR5.6. *Ter* sites were inserted with an efficiency of 53/65 (81.5%—excluding integrations attempted for the insertion of *TerB* in the non-permissive orientation). It is important to note that *TerB* in non-permissive orientation could not be inserted using either a TargeTron or the Lambda Red recombination system [30]. All viable strains reached the same plateau at the same time as the control strain (Figure S1) suggesting that *TerB* in permissive orientation as well as *TerH* and *TerJ* in either orientation do not impact replication forks or chromosomal segregation, which is in partial agreement with previous genomic region inversion data [29]. Furthermore, no significant difference in bacterial growth rates or delays were observed between these and the control strain. We conclude that the site-specific insertion of an ectopic *TerB* in non-permissive orientation in a strain carrying the wild type *tus* gene is unviable as a result of efficient replication fork arrest 930 kbp downstream to *oriC.* We presume that the resulting fork stalling or reversal induced by *TerB* in non-permissive orientation cannot be resolved, even in a strain with wild-type homologous recombination function such as BL21(DE3) [31].

Our data support the notion that *TerH* and *J* do not arrest nor pause replication forks and, as such, we propose to reclassify them as pseudo-*Ter* sequences. Furthermore, in light of our and previous data [5,6,18], *TerI,* which forms a faster dissociating Tus–*Ter*–lock complex (t_1/2_ = 196 s) than *TerJ* (t_1/2_ = 332 s) at 250 mM KCl [5,17], can also reasonably be dismissed as a pseudo-*Ter* site.

### 2.2. Chromosomal Binding of Tus

**Figure 2:**
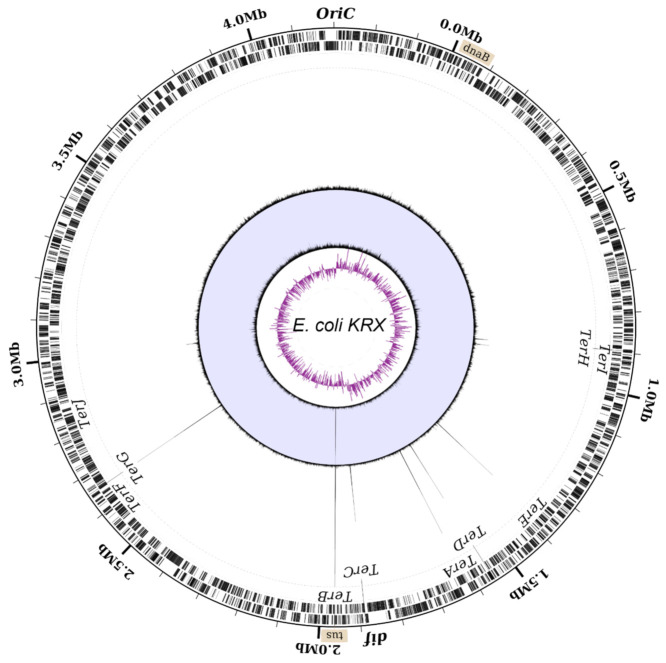
Circular representation of *E. coli* KRX chromosome with mapped ChIP-Seq coverage. From the outside to the centre of the circle: labelled forward and reverse genes; genomic location of sites and genes involved in DNA replication termination; combined ChIP-Seq read coverage (max = 430 reads at *TerB*), Input DNA read coverage (max = 230 reads at the *tus* gene), GC-skew over a 5000 bp moving window. The GC-skew switches polarity at the replication origin and terminus.

The genome wide distribution of Tus was examined by using chromosome immunoprecipitation (ChIP)-Seq and ChIP-qPCR (Figure S2) to identify the *Ter* sites that are bound during active DNA replication. Due to the low natural abundance of Tus and the unavailability of Tus-specific antibodies, the chromosomal distribution of GFP-tagged Tus (Tus-GFP) was examined in exponentially growing *E. coli* (KRX). Tus autoregulates its expression via binding to *TerB* located within the promoter region of the chromosomal *tus* gene [23,24]. As such, we hypothesised that in the presence of excess Tus-GFP, the transcription of the *tus* gene would be downregulated further. This would have the effect of reducing in vivo Tus levels, allowing excess Tus-GFP to efficiently compete for *Ter* sites. Due to the unique base sequences flanking each *Ter* site, ChIP samples could be sequenced using 50 bp Illumina reads, thereby ensuring that the reads containing the full or partial 23 bp *Ter* sequences could be accurately mapped to the genome. Input and immunoprecipitated DNA samples were sequenced and the reads mapped back to our KRX genome assembly to generate a high-resolution genome-wide distribution map of Tus-GFP (Figure 2).

A large peak at the *tus* locus was clearly visible in the input DNA sample, which corresponded to the plasmid-encoded *tus* sequence counts. The coverage at *tus* in the input DNA indicated a plasmid copy number per chromosome of ~ 43. ChIP-Seq peaks were immediately apparent without the need for a peak identification workflow. Six large peaks were visible in the immunoprecipitated coverage plot, corresponding to the binding of Tus-GFP to individual *Ter* sites. We inspected the read coverage in the 10 bp region between the chromosomal *tus* and *TerB* loci to ensure that the reads originating from plasmid-encoded *tus* did not bias the read count at *TerB* (Figure S3). The base coverage at *TerB* is equivalent to the average chromosomal reads in the input DNA, demonstrating that our method does not lead to a coverage bias in the immunoprecipitated DNA data. The average coverage values ranged from ~5 at *oriC* to ~1 in the terminus region, indicating that at least three replication forks were progressing on each chromosome arm towards the terminus region. Our ChIP-seq data revealed that out of the 10 primary *Ter* sites, only the 6 high-affinity *TerA-E and G* sequences [5] are significantly bound by excess Tus-GFP (Figure 2 and Figure S4).

Surprisingly, despite being the major termination site [6], *TerC* was one of the least bound in this group with an average 269 × read coverage compared to 430 × coverage at *TerB*. The coverage at *Ter*G (410 ×) was similar to *TerB*, suggesting that this site is almost certainly bound at normal bacterial Tus concentrations. Given the strong Tus binding and lock-forming ability of *TerG* [5], our data suggest that the absence of paused fork intermediates in the fork arrest assay measured by Duggin and Bell [6] is a result of the replication fork not reaching this *Ter* site. As anticipated, no binding was observed at the pseudo-*TerF* [4,5,6,32]. Out of the three moderate *Ter* sequences, *TerJ* in its locked complex with Tus is the most stable with respect to t_1/2_ [5] and fork arrest activity [6], yet no peak was observed, strongly supporting our ectopic insertion data and that the latter is also a pseudo-*Ter* site in natural conditions. *TerH* and *TerI* sites have similar coverages (57 × and 48 ×, respectively) corresponding to only 11–13% of the coverage at *TerB*, despite the bacterial Tus-GFP concentration being 1000-fold higher than the normal endogenous levels of Tus, suggesting these sites would be mostly unbound at normal cellular Tus concentration. Taken together with previous affinity data, our ChIP-Seq and ectopic insertion findings support the notion that *TerH*, *I* and *J* do not have a role in replication fork arrest. Our ChIP-Seq dataset was confirmed by ChIP-qPCR (Figure S5) and allowed delineation of a refined minimal replication fork trap within *E. coli* comprising two clusters of three *Ter* sites: (a) *TerB*, *C* and *G* that can arrest a clockwise moving replication fork and (b) *TerA*, *D* and *E* that can arrest an anticlockwise moving replication fork (Figure 2).

### 2.3. GC-Skew Relative to Termination Site Usage in E. coli

Although the GC-skew is a well-recognised tool to identify the origin of replication in many circular prokaryotic chromosomes [33,34,35], the feasibility of utilising the GC-skew to predict the terminus has been debated amongst researchers due to the terminus shift point being closer to the chromosome dimer resolution site (*dif*) than to the *Ter* sites in some studied species [36]. However, the GC-skew has recently been shown to coincide with replication fork arrest by Tus at *Ter* sites and is not influenced by *dif* [37,38]. We hypothesised that the GC-skew should correlate with the frequency of fork arrest activity at specified *Ter* sites. In other words, the GC-skew is representative of the average of the ensemble of replication forks collision loci at functional Ter sites. In this scenario, the inflection point should occur at the historical positional average between the Ter sites where termination occurs. Duggin and Bell showed that only TerA, B and C have significant replication arrest activity (0.19%, 0.14% and 0.85%, respectively) in natural Tus conditions [6]. We tested this scenario and found that the expected average position of replication termination (based on the positional and fractional distribution of replication fork arrest activity) almost coincided with the GC-skew inflection point, i.e., only 7.5 kb from the calculated inflection point derived from a sliding 1000 bp cumulative GC-skew (Figure S6). It is important to note that the *dif* site is located 8 kb from the terminal GC-skew switch point on the other chromosomal arm. We tested additional scenarios but none produced a better correlation. Taken together with previously published data [37,38], the GC-skew of *E. coli* supports the involvement of *TerA*, *B* and *C* in replication fork arrest and provides an invaluable tool to further our understanding of replication termination in other species.

### 2.4. A Narrow Fork Trap Dyad in Edwardsiella Tarda

**Figure 3:**
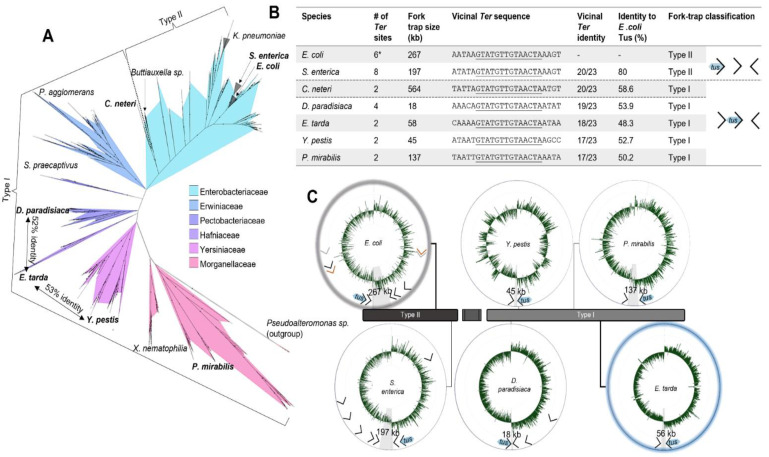
Phylogenetic analysis of Tus orthologs and fork trap architecture in Enterobacterales. (**A**) Unrooted phylogenetic relationship of ~2500 Tus protein sequences using InterPro entries (IPR008865) highlighting the transition of a simple type I to complex type II fork trap architecture, which occurs at *Cedecea*. (**B**) Chromosomal fork trap characteristics and classification for selected species (see Figure S7 and Table S1 for their graphical representations and the complete table of species). Fork trap size (kb) corresponds to the distance between the two innermost *Ter* sites of opposite polarity. Underlined bases represent a continuous identical sequence shared between all *Ter* sequences vicinal to *tus* starting at the GC(6) base-pair. *: excluding pseudo-*Ter* sites. (**C**) The different types of replication fork trap architecture in Enterobacterales.

While the function of *TerA*, *B* and *C* and their replication fork arrest activity in *E. coli* is clear, the need of *TerE*, *D* and *G* is not, despite their high affinity for Tus and ability to form a Tus–*Ter*–lock [5]. While trying to gain further insight into these seemingly redundant *Ter* sites, we examined the replication fork trap architecture in closely, moderately as well as distantly related bacteria harbouring a *tus* gene (Figure 3A,B). A recent phylogenetic analysis of Tus homologs in bacteria identified resident *tus* genes within the chromosomes of most Enterobacterales [39]. Using a streamlined approach, we characterised the replication fork traps in several of these species (Figure S7 and Table S1). Following identification of the vicinal *Ter* sequences upstream of *tus* genes in our selected bacterial genomes, BLAST searches were performed to identify other *Ter* sites within the genomes as well as their replication fork blocking orientations. The stringency of our approach was evaluated with *E. coli* K12, identifying all primary *Ter* sites as well as the pseudo-*TerF*, *H*–*J* but excluding the pseudo-*TerK*, *L*, *Y* and *Z.*

In *Salmonella enterica*, a close relative of *E. coli*, the left chromosomal arm contains five *Ter* sites, while the right chromosomal arm contains only three *Ter* sites in opposite orientation (Figure S7A). The Tus protein and vicinal *Ter* sequence identities (i.e., corresponding to the *E. coli* Tus protein and *TerB*) were found to be 80% and 87%, respectively. The distance between the innermost *Ter* sites (197 kb) is significantly reduced in the *Salmonella* replication fork trap. In more distantly related bacteria, such as *Dickeya paradisiaca* and *Proteus mirabilis*, despite the high sequence identity of their respective *Ter* sequences vicinal to *tus* (83% for both), a reduction in the number of *Ter* sites as well as a narrowing of the fork trap (i.e., the distance between the innermost *Ter* sites) were commonly seen (Figure 3B). Most striking was that the innermost *Ter* site upstream of the *tus* gene (i.e., corresponding to the *E. coli TerC*) was no longer present in these species (Figure 3B). To our surprise, all replication fork traps that we characterised outside the Enterobacteriaceae family lacked the innermost *Ter* site corresponding to the *TerC* in *E. coli*. In these genomes (*D. paradisiaca*, *P. mirabilis*, *X. nematophila*, *E. tarda* and *Yersinia pestis*), the innermost *Ter* site is the one vicinal to the *tus* gene in that cluster (Figure S7 and Table S1). We thus propose a new replication fork trap classification based on their architecture where a type I replication fork trap has one of its innermost *Ter* sites vicinal to *tus* (Figure 3B). Accordingly, *E. coli* and *S. enterica* genomes contain type II replication fork traps where *Ter* vicinal to *tus* is not in an innermost position. All type I replication fork traps that we identified outside of the Enterobacteriaceae family are significantly narrower than the type II traps (Figure 3B). This is most evident in *D. paradisiaca* for which the innermost *Ter* inter-distance is just 18 kb.

**Figure 4:**
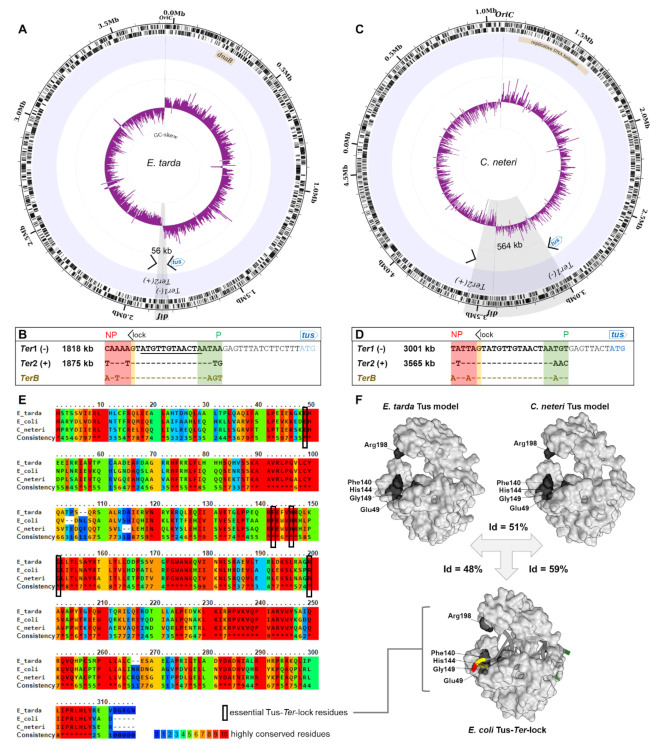
Prototypical type I replication fork trap. (**A**) Circular representation of *E. tarda* (strain EIB202) chromosome. Illustrated from the outside to the centre of the circle: forward and reverse genes, labelled genomic location of identified *Ter* sites involved in DNA replication termination, simplified annotation of the termination fork trap utilised, GC-skew over a 5000 bp moving window. The sharp GC-skew switches polarity at the replication origin and between the two identified *Ter* sites near *dif*. (**B**) Sequence alignment and genomic locations of the *E. tarda Ter* sites and *TerB* from *E. coli*. *Ter1* is located slightly upstream of the start site (ATG) of the *tus* gene similar to *TerB* in *E. coli.* The strictly conserved 12 bp core sequence is underlined and the G(6) base complementary to C(6) is highlighted in yellow. NP: non-permissive face (red), P: permissive face (green). (**C**) Circular representation of *C. neteri* (strain ND14a) chromosome. (**D**) Sequence alignment and genomic locations of the *C. neteri Ter* sites and *TerB* from *E. coli*. (**E**) Tus protein sequence alignment (PRALINE) with highlighted conserved residues. (**F**) Comparison of the *E. coli* Tus–*Ter*–lock complex 3D structure (PDB 2I06) and the modelled structure of *E. tarda* and *C. neteri* Tus proteins using SWISS-MODEL. The essential amino acid residues in the cytosine binding pocket are indicated. The theoretical isoelectric points of *E. coli*, *E. tarda* and *C. neteri* Tus are 9.57, 9.67 and 9.31, respectively.

In *Edwardsiella tarda*, this pattern of simplification culminated into a narrow and perfectly symmetrical replication fork trap diametrically opposite the *oriC*, consisting of two unique *Ter* sequences (Figure 3C). *E. tarda Ter1* and *Ter2* are only 56 kb apart and equidistantly located on either side of the hypothetical terminus site (Figure 4A). The next *Ter*-like sequence within this genome has only 65% identity to *Ter1* with a high level of degeneracy in the core sequence (see pseudo-*Ter3* in Figure S7F) and would oppose an o*riC*-initiated replication fork. Most importantly, the midpoint between *Ter1* and *Ter2* (~1846 kb, *cf*Figure 4B) coincides almost perfectly with the sharp GC-skew flip (~1847 kb) and *dif* (~1843 kb), suggesting that they are being used equally as replication fork barriers. E. tarda *Ter1* shares 78% sequence identity to *E. coli TerB* (Figure 3B). In contrast, *E. coli TerC* shares only 74% sequence identity to *TerB*, immediately suggesting that the mechanism of polar DNA replication fork arrest in *E. tarda* also involves formation of a locked Tus–*Ter* complex.

In our efforts to investigate the evolutionary divergence of the type I/II fork traps, we identified a unique group of *Cedecea* species within the Enterobacteriaceae family that uses a type I replication fork trap system with only two oppositely oriented *Ter* sites for *C. neteri* (Figure 3B and Figure 4C,D). Against our expectations, the fork traps within this rare genus of bacteria were the widest (507–564 kb) of all investigated bacteria and the GC-skew switch (~3420 kb) in *C. neteri* fitted unambiguously with the *dif* location (~3424 kb). Of note, the *dif* site and GC-skew are located diametrically opposite the *oriC*, while the fork trap consisting of *Ter1* (3001 kb) and *Ter2* (3565 kb) is not.

While all vicinal *Ter* sequences that we examined are highly homologous (74–83% identity to *E. coli TerB)* and include the crucial C(6), and there is little doubt that the Tus orthologs from *S. enterica*, *Y. pestis* and *P. mirabilis* are able to arrest a replication fork at *TerB* [40], the competency of *C. neteri, D. paradisiaca*, *X. nematophila* and *E. tarda* Tus orthologs (46–59% identity to *E. coli* Tus) to form a locked complex is not clear (Figure 3B). To examine if these Tus orthologs are competent in forming a locked complex, we verified that the residues that make a critical interaction with the C(6) base are strictly conserved (Figure 4E). It is apparent that *C. neteri*, and especially *E. tarda* Tus, despite having one of the lowest identity scores with the *E. coli* Tus sequence (48%), should be fully competent in forming locked complexes with their *Ter* sequences. Furthermore, model structures of *C. neteri* and *E. tarda* Tus showed no major differences in their respective cytosine binding pockets when compared to *E. coli* (Figure 4F), supporting the formation of a highly efficient Tus–*Ter*–lock complex in both species. The sharp GC-skew flip midway between *Ter1* and *Ter2* suggests that replication forks rarely break-through the fork trap dyad in *E. tarda*. However, in *C. neteri*, it seems rather unlikely that *Ter1* and *Ter2* are being utilised to arrest replication forks.

## 3. Discussion

### 3.1. A Simplified Type II Replication Fork Trap in E. coli

Since the discovery of the first *Ter* sites and Tus coding sequence in *E. coli*, additional *Ter* sites were identified, simultaneously expanding the size of the replication fork trap and increasing the perceived complexity of DNA replication termination. The systematic analyses of individual *Ter* sites both in vitro and in vivo with respect to their affinity and kinetics for Tus, ability to form a Tus–*Ter*–lock structure as well as their position and orientation within the bacterial genome have provided a wealth of information as to how this seemingly simple protein–DNA interaction impedes replication forks. In fact, Tus–*Ter* has become one of the best-understood protein–DNA complexes, leading to the development of a variety of biotechnological applications [41,42,43,44]. Yet, we are only just starting to understand the modus operandi of Tus in vivo. Duggin and Bell showed evidence of a simple replication fork trap involving just *TerA, B* and *C* under normal bacterial concentrations of Tus [6]. We found that the observed distribution of fork arrest events at these sites fits with the terminus GC-skew flip in the *E. coli* genome. This is in agreement with Duggin and Bell who found no evidence supporting site-specific termination at or near the *dif* site [6], despite it being located only 8 kb from the terminal GC-skew switch point.

Taken together, our findings allow us to propose a simplified replication fork trap in *E. coli* consisting of just six *Ter* sites (three in each cluster) and support the notions that: (i) Tus binds preferentially to the high-affinity *Ter* sites in vivo; (ii) Tus-bound *TerC* and *TerA* are sufficient to block replication forks progressing towards their non-permissive face; (iii) *TerB* is most likely only used when a replication fork passes through an unbound *TerC*; (iv) replication forks are unlikely to reach the outer *Ter* sites; (v) *TerH, I* and *J* are unlikely to be bound at natural Tus concentrations, are unable to block replication forks and, thus, cannot be considered as functional *Ter* sites.

While the roles of *TerA*, *B*, and *C* are now clear, the need for *TerD*, and particularly the distant *TerE* and *TerG* in the *E. coli* genome, remains somewhat enigmatic. If we consider that a single genomic insertion of *TerB* in the non-permissive orientation at SIR5.6 is not viable despite being the furthest from *tus* and the low natural abundance of Tus [45], this would support the notion that *TerD, E* and *G*, although bound by Tus, are rarely used to arrest replication forks. The distribution of Tus to these *Ter* sites is rather unexpected when considering the distribution of replication fork pausing events reported by Duggin and Bell [6], yet it fits well with previously published affinity data [4,5,17].

### 3.2. The Ancestral Type I DNA Replication Fork Trap

The *E. coli* Tus–*Ter*-mediated replication fork arrest mechanism has been intensely scrutinised to better understand the final step in bacterial DNA replication. However, it now appears that the type II replication fork trap, which is mostly found in Enterobacteriaceae, is more of an exception or even an anomaly with respect to its many redundant *Ter* sites and their wide spread around the chromosome. It seems that the complexity of the type II fork trap in *E. coli* and closely related species [46] has merely distracted scientists from capturing the elegance and simplicity of the type I system in other Enterobacterales. Nevertheless, the work on the *E. coli* Tus–*Ter* complex was instrumental to decipher the unique Tus–*Ter*–lock mechanism [9], which still stands true and is seemingly conserved in all *tus*-harbouring bacteria.

Here, we show that the architecture and complexity of replication fork traps vary significantly across *tus*-harbouring bacteria. Yet, the two distinct classes of fork traps contain highly conserved *Ter* sequences despite moderate identity scores between Tus sequences. In the narrow type I fork trap, the *Ter1* vicinal to *tus* acts as a primary *Ter* site to arrest an incoming DnaB helicase travelling toward its non-permissive face (Figure 3B). The sharp terminal GC-skew switch observed in *E. tarda* strongly suggests that replication forks do not break through the *Ter* sites, and advocates against the need for redundant back-up *Ter* sites. We propose that the narrow *E. tarda* fork trap consisting of only two *Ter* sites diametrically opposite of the *oriC* represents a perfectly positioned ancestral type I replication fork trap that is evolutionarily stable (Figure 4A).

**Figure 5:**
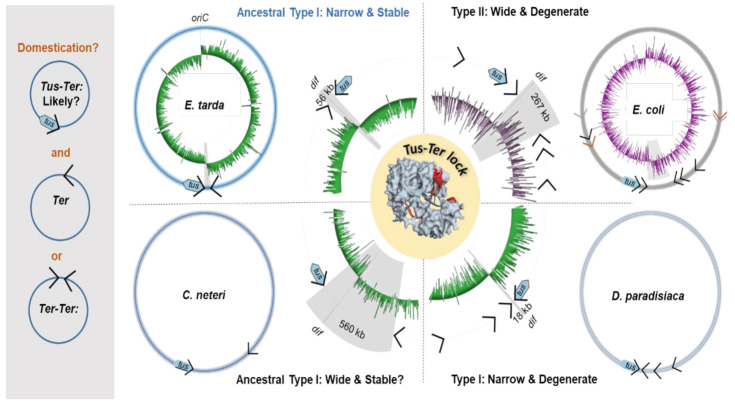
Ancestral domestication of Tus and *Ter* suggesting possible routes to the different replication fork trap architectures. Adoption of a wide ancestral replication fork trap (such as observed in *C. neteri*) that is sub-optimally located for termination may lead to the degeneracy and redundancy of *Ter* sites, and increased fork trap complexity observed in *E. coli* and *D. paradisiaca*.

The unusually wide and ill positioned type I fork trap discovered within *Cedecea* species (Figure 4C) also seems to be evolutionarily stable. Yet, it is unclear whether replication forks would even reach a blocking Tus–*Ter* complex in these bacteria, as the GC-switch occurs diametrically opposite to the origin at the *dif* locus (Figure 4C). This wide fork trap immediately suggests a possible route to the more complex replication fork traps observed within Enterobacteriaceae (Figure 5). As such, it is possible that upon domestication of a wide and probably ill placed fork trap, the large distance between *Ter1* and *Ter2* could be a selective driver for acquiring an additional *Ter* site closer to the terminus and *dif* site rather than the inherent need for a back-up system due to the inefficient Tus–*Ter*–lock formation that has previously been reported in *E. coli* [13,14,15].

### 3.3. Conclusions and Perspective

Our in silico data support the notion that in bacteria harbouring a type I fork trap, the Tus–*Ter* interactions are competent in arresting an incoming replication fork via Tus–*Ter*–lock formation. Furthermore, the ancestral type I system clearly demonstrates that there is no inherent requirement for a back-up system to trap replication forks in the terminus region. However, what is the consequence of the absence of back-up *Ter* sites on lock strength and protein–DNA binding affinity in these systems? Examination of Tus–*Ter* found in type I fork traps will certainly help answer this important question and delineate the essential features and requirements of these unique protein–DNA interactions.

The diversity of type I and type II fork traps with respect to the number of *Ter* sites and their narrow or wide distribution begs the question as to what the evolutionary drivers for such variety are (Figure 5)? In *E. coli*, the distance of *TerB* from the terminus is not optimal for efficient replication termination and, thus, an additional *TerC* site with increased fork arrest activity was advantageous in narrowing the fork trap near the *dif* site. The discovery of the extremely wide and somewhat ill positioned type I fork traps found in *Cedecae* species would support this hypothesis (Figure 5).

The existence of both very narrow and very wide replication fork traps that are evolutionary stable is particularly puzzling. Indeed, the wide *Cedecae* fork trap could suggest that here, replication stalling activity may not be a primary function. Comparative examination of narrow and wide fork traps could be key to fully decipher the mechanism of DNA replication termination and particularly the intersection between *dif* sites and fork traps as well as possible additional roles of Tus–*Ter*, e.g., in chromosomal segregation [22].

In recent years, investigations into the *E. coli* Tus–*Ter* interaction have shifted towards technology development. We anticipate that our Tus–*Ter* data will generate an impetus to develop new molecular tools. Indeed, orthologous Tus–*Ter* systems with different *Ter* binding-affinities would be very useful to develop finely tuneable assays to study DNA replication and transcription perturbation effects [41] and other biotechnologies [4,43]. Lastly, we also anticipate that the delineation of the ancestral replication fork trap unveiled in this work will finally simplify the teaching of bacterial DNA replication termination in undergraduate molecular biology courses.

## 4. Materials and Methods

A detailed list of materials and other resources is available in the supplementary data. ChIP-Seq and genome data have been deposited in Gene Expression Omnibus (GEO) with the accession number GSE163680.

### 4.1. Plasmids and Protein Expression

A pET-based vector encoding His_6_-Tus-GFP (pPMS1259) was used for protein expression with ampicillin selection [32,47,48,49]. *E. coli* KRX (Promega), which is a K12 derivative, was used to express His_6_-Tus-GFP for ChIP experiments. His_6_-Tus-GFP proteins used as protein standards and controls were expressed in *E. coli* BL21(DE3)RIPL under autoinduction conditions and Ni-affinity purified as previously described [32], and stored in 50 mM sodium phosphate (pH 7.8) and 10% glycerol (*w*/*v*).

### 4.2. Protein Expression and Crosslinking

Competent *E. coli* KRX bacteria were transformed with pPMS1259, plated onto LB plates supplemented with ampicillin (100 µg/mL) and grown overnight at 37 °C. Colonies were resuspended and diluted to an OD_600_ of 0.1 in 12 mL of LB broth supplemented with ampicillin (100 µg/mL). Cultures were grown for 45 min at 37 °C before inducing moderate levels of His_6_-Tus-GFP with 0.02% Rhamnose (*w*/*v* final culture concentration). Cultures were incubated for another 2 h at 37 °C, followed by 2 h at 16 °C. Culture aliquots (9 mL) were cooled on ice for 30 min to which 250 µL of a formaldehyde solution (36% *w*/*v*) was added (final concentration of 1%). The bacterial suspensions were then placed at room temperature for 20 min. Glycine powder was added the bacterial suspensions (0.5 M final concentration) for 5 min at room temperature followed by 5 min on ice. The bacterial suspensions were centrifuged for 5 min at 800× *g* and 4 °C and washed twice with 4 mL and 10 mL of cold TCS buffer (50 mM Tris (pH 7.5), 150 mM NaCl and 2 mM KCl). The supernatants were discarded and the bacteria pellets were stored at −80 °C until required. KRX bacteria (without plasmid) were subjected to the same protocol and used as control.

### 4.3. Detection and Quantitation of GFP-Tagged Protein Expression

Culture aliquots were taken prior to crosslinking, centrifuged at 1000× *g* for 1 min and the pellets were resuspended in 2X Laemmli buffer at a concentration of 7.8 × 10^9^ bacteria per mL. The suspensions were heated for 10 min at 90 °C and 5 µL (corresponding to the total protein content of 3.95 × 10^7^ bacteria) was separated in a 10% SDS-polyacrylamide gel alongside 0.5 µg of reference His_6_-Tus-GFP protein standard for Western blot analysis. Chicken anti-GFP IgY (Abcam ab92456) and HRP-conjugated goat anti-IgY (Jackson 103-035-155) were revealed with SIGMAFAST™ 3,3′-Diaminobenzidine tablets. Protein bands were quantified using ImageJ (http://rsbweb.nih.gov/ij/ (accessed on 14 December 2021)) and intracellular concentrations were estimated based on the intensity of bands of known protein concentration and using cell parameters determined by Volkmer and Heinemann for cell volume (4.4 fl) and cell concentration at a given OD_600_ in LB (i.e., OD_600_ of 1 corresponding to 7.8 × 10^8^ bacteria per mL) [50]. 

### 4.4. Chromosome Immunoprecipitation

Bacteria pellets were resuspended in lysis buffer (10 mM Tris (pH 8), 20% sucrose, 50 mM NaCl, 10 mM EDTA, 1 mg/mL lysozyme and 10 µg/mL RNase) in 1/10 of initial culture volume (adjusted between replicates to reach same suspension concentration). Following a 30 min incubation period at 37 °C, the lysates were diluted 5 times in IP buffer (50 mM HEPES-KOH (pH 7.5), 150 mM NaCl, 1 mM EDTA) and passed three times in a French press at 12,000 psi to ensure maximum and reproducible cell lysis and DNA shearing. The Tus-GFP lysates were heated for 10 min at 50 °C to denature free Tus-GFP [5,32,48]. Control KRX lysates were processed identically. All lysates were centrifuged at 30,000× *g* for 20 min at 4 °C. Supernatants were used for immunoprecipitation and as input samples. For immunoprecipitation, 96-well MaxiSorp round bottom U96 Nunc plates were coated overnight at 4 °C with 50 µL of 50 mM sodium phosphate (pH 7.5) and 10% glycerol buffer containing 0.5 µg of goat anti-GFP IgG (Abcam; Ab6673). Wells were washed once with 200 µL of TCS buffer prior to immunoprecipitation. After the wash step, 50 µL of lysate supernatant was added per well for 90 min at room temperature. Wells were washed three times with 200 µL of TCS buffer. Control immunoprecipitation experiments were performed in parallel without antibody pre-coating as background controls. Immunocaptured DNA was released by adding 50 µL of elution and de-crosslinking buffer (2 mM Tris (pH 7.5), 50 mM NaCl, 0.005% tween and 300 µg/mL proteinase K) to each well for 1 h at 37 °C (output). Input samples were diluted 10,000 times in elution buffer (2 mM Tris (pH 7.5), 50 mM NaCl, 0.005% Tween) and 50 µL was transferred to a tube containing proteinase K, yielding a final concentration of 300 µg/mL to de-crosslink the input DNA for 1 h at 37 °C. Samples (inputs and outputs) were incubated 15 min at 95 °C to denature proteinase K and residual crosslinked proteins. After 5 min incubation on ice, samples were centrifuged at 18,000× *g* for 5 min at 4 °C and the supernatants were used for qPCR and Illumina sequencing.

### 4.5. qPCR Protocol 

All qPCR reactions were performed as previously described [4]. Oligonucleotides for amplification of *oriC* and *Ter* containing regions are listed in Additional Resources. Briefly, qPCR reactions contained 2 µL of input or output DNA sample, 8 µL of primer pairs (0.5 µM each) and 10 µL of SensiMix SYBR and fluorescein mastermix (Bioline). The protocol included a 10 min step at 95 °C followed by 40 cycles at 95 °C for 10 s and 60 °C for 15 s. Melt-curves were run for quality control. *Ct* values were obtained at a set threshold applied to all experiments. Standard curves were performed in triplicate with purified and serially diluted *Ter* and *oriC* amplicons in matching output buffer conditions. For each primer pair, the average slope value of three standard curves (*n* = 3) was used to determine the primer-specific amplification efficiency according to the following equation [51].

$$E_{amp}=10^{\left(-\frac{1}{\mathrm{slope}}\right)}$$

A melt-curve was performed to verify that the correct regions were amplified.

### 4.6. qPCR Analyses

For all qPCR experiments, *Ct* values were determined at the same threshold value. *Ct*_(*input*)_ values were corrected for the dilution factor to give *_c_Ct*_(*input*)_ according to the following equation:

Cctinput=Ctinput−logEampdilution factor

The immunoprecipitation efficiency of each specific target DNA region relative to a non-specific DNA region (*IP efficiency*_(*oriC*)_) was calculated according to the following equation:

IP efficiencyoriC=EampCctinputsp−CtoutputspEampCctinputns−Ctoutputns

where *_c_Ct*_(*input*)_ and *Ct*_(*output*)_ are values obtained for each DNA target before (input) and after ChIP (output). Specific DNA target (i.e., *Ter* sites) and non-specific control DNA region are indicated with “sp” and “ns” subscripts, respectively.

### 4.7. Library Preparation and Illumina Sequencing

Input and output DNA samples were purified using Wizard PCR clean up and eluted in 110 µL water. Each library was prepared using the NEBNext Ultra DNA library preparation kit for Illumina. ChIP output samples (~0.25 ng) were used for library preparation. The libraries were prepared according to the manufacturer’s instructions. Briefly, DNA suspensions (55.5 µL) were end-repaired. Due to the low DNA concentrations in the suspensions, the NEB adaptors were diluted 10-fold in water to 1.5 µM for ligation as recommended. The adaptors were cleaved using uracil excision. Size selection was not recommended for samples < 50 ng. DNA was then cleaned up using Sera-Mag beads (ratio of 1.4) and eluted in 28 µL of 0.1 × TE. Index primers were added by PCR using 18 cycles. (13–15 cycles were recommended for 5 ng of input material; therefore, 3 cycles were added to account for the 20-fold difference in input DNA). DNA quantification was performed using the Quantifluor dsDNA system (Promega). The samples were pooled in a single library, denatured and loaded for sequencing with an Illumina MiSeq desktop sequencer (50 bp single-end sequencing). Illumina read quality was assessed using FastQC (v.0.11.8) followed by removal of Illumina adapters and leading and tailing nucleotides with a Phred score ≤ 10 over a 6 bp window using Trimmomatic (v.0.36).

### 4.8. DNA Preparation for Nanopore Long Read Sequencing

A flask containing 10 mL of LB media was inoculated with 100 μL of KRX *E. coli* overnight culture. The culture was incubated at 37 °C and 150 RPM until log phase was reached (OD_600_ = 0.7) at which point chloramphenicol was added at a final concentration of 180 μg/mL to inhibit protein synthesis. The bacteria were centrifuged and resuspended in 3.5 mL lysis buffer (114 mM Tris-HCl (pH 8), 115 mM EDTA, 570 mM NaCl and 1% triton X-100). After the addition of lysozyme (250 μL, 50 mg/mL), sodium dodecyl sulphate (500 μL, 10% *w*/*v*) and RNase A (4 μL, 100 mg/mL), the bacterial suspension was inverted gently 12 times and heated at 50 °C for 30 min. Proteinase K (500 μL, 10 mg/mL) was added with repeated gentle mixing and heating steps. The suspension was then combined with 8 mL precipitation buffer (75% isopropanol, 2.5 mM ammonium acetate), inverted gently 15 times followed by centrifugation at 4000× *g* at 4 °C for 5 min. The supernatant was discarded. The soft DNA precipitate was transferred using a wide bore tip into a 1.5 mL tube, resuspended with 70% ethanol and stored at 4 °C for 5 min, then centrifuged and the supernatant removed. The DNA pellet was air dried for 5 min then resuspended in 200 μL nuclease-free water and heated at 50 °C for 5 min with the tube cap open. The DNA was sheared using a syringe with a 20-gauge needle (3 times). DNA concentration and quality were assessed using Invitrogen Qubit 4 and agarose gel electrophoresis.

### 4.9. Nanopore Long Read Sequencing and Genome Assembly

A Nanopore sequencing library was prepared using the Rapid Sequencing protocol SQK-RAD004 (Oxford Nanopore). As recommended by the protocol, 7.5 µL of DNA suspension (400 ng) was added to the flow cell. Sequencing was performed on a FLO-MIN106 R9 MinION flow cell. Base-calling was processed using the pipeline implemented in MinKNOW software version 18.01.6 (Oxford Nanopore). In total, 1.17 GB (253 × coverage) of sequence data was generated for *E. coli* strain KRX over ~18 h of sequencing achieved in two separate runs. Prior to assembly, all fastq files were combined and quality filtered by nanofilt version 2.5.0 (quality score ≥ 9). The remaining ~226 thousand reads had an average length of 3738 nucleotides with the longest read of ~180 thousand nucleotides and a total 146 × coverage of the KRX *E. coli* genome. Oxford Nanopore adapters were trimmed using Porechop (version 0.2.3_seqan2.1.1) and assembled using Flye (version 2.6) with default settings for Oxford Nanopore data. Polishing was performed with Racon iteratively four times in combination with Pilon (version 1.23-1) using the entire Illumina ChIP-Seq data (i.e., KRX Input, WGS, negative control and ChIP DNA). The genome was annotated using Prokka (version 1.14.0) and evaluated using Quast (version 5.0.2) against the *E. coli* K12 genome (GenBank assembly accession: GCA_000005845.2).

### 4.10. ChIP-Seq Analysis

Following adapter trimming and quality control, each biological and technical replicate fastqc file of sequenced samples (i.e., Input, ChIP DNA and negative control) was individually aligned to the polished KRX genome using Bowtie2 (version 2.3.4.1). Samtools (version 1.9) was then used to organise each alignment file for visualisation on Interactive Genomics Viewer (IGV) to visually assess the data in terms of replicability between each replicate and for any outliers. The replicate data for Input, ChIP and negative control were pooled into three separate fastqc files and aligned to the KRX *E. coli* genome using the default settings of Bowtie2. A circular annotation of each pooled ChIP-Seq reads mapped to the KRX *E. coli* genome spanning a 23 bp window (size of an extended *Ter* site) was created as well as the GC-skew of the chromosome (5000 bp window) using Circleator (version 1.0.2). The individual positions and orientations of *Ter* sites were identified and verified using their specific 23 bp sequences and compared to the respective sites in *E. coli* (K12). The single base read counts were averaged over the 23 base *Ter* sequences for Input, ChIP and negative control DNA using genomeCoverageBed (Version: v2.26.0).

### 4.11. Genome Engineering of Ectopic Ter Sites

Targetrons (mobile group II introns) were designed to insert *Ter* sites in the safe insertion region SIR.5.6 [52], located in the right non-structured chromosome domain (Figure 1A) of *E. coli* BL21(*DE3*) (accession number AM946981). The SIR5.6 is located about 930 kbp downstream of *oriC* (right chromosome arm). *Ter*-targetrons (mobile group II introns carrying *Ter* sequences) insertion was performed as described previously [25], replacing *lox* sites with *TerB*, *TerH* or *TerI* sequences in permissive or non-permissive orientations. Insertion of *TerB* in the non-permissive orientation was also attempted using the Lambda Red recombination system but no viable colonies could be obtained. The successful insertions of the other *Ter* sites were confirmed by colony PCR and verified by sequencing. *Ter* sites were inserted into SIR5.6 with an efficiency of 53/65 (81.5%—excluding integrations attempted for the insertion of *TerB* in the non-permissive orientation).

BL21(*DE3*) cells carrying ectopic *Ter* sites were grown in LB broth at 37 °C and OD_600_ was measured every 5 min for 12 h. The results were plotted as log_2_(OD_600_) versus time (minute). In order to select the linear region of the curve, each point was assigned a correlation coefficient R^2^ corresponding to the value of R^2^ for the line consisting of that point and the five points before and after. The variance was lower when the same time window was used for all three replicates so the resulting R^2^ values were averaged for all three replicates at each time point. The longest stretch in which all these averaged R^2^ values were equal to or greater than 0.99 was taken as the linear range. The slope of the least-squares linear fit of the log_2_(OD_600_) curve of each replicate in that time range was then taken as the growth rate and the doubling time was calculated as 1/growth rate.

### 4.12. Fork Trap Characterisation in Enterobacterales 

Bacterial species containing a *tus* gene ortholog were identified using InterPro entry IPR008865 including only the entries for Enterobacterales (2518 protein sequences in total). Several Tus protein sequences from a selection of *Pseudoalteromonas* species were also included as the outgroup. Ten rounds of an alignment for all sequences were generated using MUSCLE software with default settings [53]. The tree was constructed using the matrix of aligned sequences in RAxML v8.2.0 [54], which performed an ML phylogenetic analysis with 100 independent repetitions using the PROTGAMMALGI molecular evolution model in combination with an independent rapid bootstrap algorithm (--AutoMRE) to establish support for each node. The consensus tree produced by RAxML was visualised and edited in iTol [55]. The sequences that branched off earlier than the outgroup were removed from the final tree as these were most likely incorrectly assigned taxa or sequences that are most likely not Tus protein sequences. For clarity, clades from *Escherichia coli*, *Salmonella* and *Klebsiella* were collapsed due to the large number of sequences. 

A random selection of species from different families were chosen including *Dickeya paradisiaca* (strain Ech703), *Edwardsiella tarda* (strain EIB202), *Yersinia pestis* (Microtus str. 91001), *Xenorhabdus nematophila* (strain ATCC 19061), *Proteus mirabilis* (strain HN2p), *Cedecea neteri* (strain ND14a) and *Salmonella enterica* serovar Typhimurium LT2. Genome assemblies that were preliminary were excluded from fork trap analysis. Upon identification of a *tus* gene ortholog, the adjacent *Ter* site was identified within its 50 bp 5’ UTR by aligning the 23 bp *E. coli TerB* sequence. For each selected species, a BLAST search was carried out using the adjacent *Ter* sequence to locate further *Ter* sites within their genome. Sequences were verified by inspecting each BLAST to ensure it contained the locking C(6) followed by the conserved 12 bp core spanning from A(8) to A(19) (Figure 1B). A circular annotation of each genome to display the architecture of the fork trap was generated alongside the GC-skew of the chromosome (5000 bp window) using Circleator (version 1.0.2).

### 4.13. Quantification and Statistical Analyses 

Statistics and number of biological and technical repeats are indicated in the relevant figure legends, tables and methods. Statistical analyses were performed using GraphPad Prism 7. Data are expressed as mean values ± SD, ± SE or ranges.

## Figures and Tables

**Table 1:** Effect of ectopic *Ter* sites on the growth rate of *E. coli* BL21(DE3).

Strain BL21(DE3)	Doubling Time T_D_ in min (SE)
*TerB* (P)	25.5 (0.4)
*TerH* (NP)	24.1 (0.1) *
*TerH* (P)	23.7 (0.6)
*TerJ* (P)	22.7 (0.3)
*TerJ* (NP)	23.7 (0.3)
Control	25.9 (0.5)

*TerB*, *TerH* and *TerJ* were inserted ~930 kp downstream of *oriC* in the permissive (P) or non-permissive (NP) orientation. A culture of wild type BL21(DE3) was grown as a control. Growth rates were determined from the slopes of the linear regressions performed between 100 and 210 min. Doubling times (T_D_) were calculated as 1/growth rates (*n* = 3, except for *TerH* (NP), *n* = 2). Standard errors (SE) are shown. * One outlier was omitted.

## Data Availability

The sequencing data presented in this study are openly available in the Genome Expression Omnibus (GEO) database with the accession number GSE163680. (https://www.ncbi.nlm.nih.gov/geo/query/acc.cgi?acc=GSE163680 (accessed on 15 December 2021)).

## Supplementary Materials

The following are available online at https://www.mdpi.com/article/10.3390/ijms222413533/s1.

## References

[1] Neylon C., Kralicek A.V., Hill T.M., Dixon N.E. (2005). Replication termination in Escherichia coli: Structure and antihelicase activity of the Tus-Ter complex. Microbiol. Mol. Biol. Rev..

[2] Berghuis B.A., Raducanu V.S., Elshenawy M.M., Jergic S., Depken M., Dixon N.E., Hamdan S.M., Dekker N.H. (2018). What is all this fuss about Tus? Comparison of recent findings from biophysical and biochemical experiments. Crit. Rev. Biochem. Mol. Biol..

[3] Xu Z.Q., Dixon N.E. (2018). Bacterial replisomes. Curr. Opin. Struct. Biol..

[4] Moreau M.J., Schaeffer P.M. (2012). A polyplex qPCR-based binding assay for protein-DNA interactions. Analyst.

[5] Moreau M.J., Schaeffer P.M. (2012). Differential Tus-Ter binding and lock formation: Implications for DNA replication termination in Escherichia coli. Mol. Biosyst..

[6] Duggin I.G., Bell S.D. (2009). Termination structures in the Escherichia coli chromosome replication fork trap. J. Mol. Biol..

[7] Bastia D., Zzaman S., Krings G., Saxena M., Peng X., Greenberg M.M. (2008). Replication termination mechanism as revealed by Tus-mediated polar arrest of a sliding helicase. Proc. Natl. Acad. Sci. USA.

[8] Kaplan D.L. (2006). Replication termination: Mechanism of polar arrest revealed. Curr. Biol..

[9] Mulcair M.D., Schaeffer P.M., Oakley A.J., Cross H.F., Neylon C., Hill T.M., Dixon N.E. (2006). A molecular mousetrap determines polarity of termination of DNA replication in E. coli. Cell.

[10] Neylon C., Brown S.E., Kralicek A.V., Miles C.S., Love C.A., Dixon N.E. (2000). Interaction of the Escherichia coli replication terminator protein (Tus) with DNA: A model derived from DNA-binding studies of mutant proteins by surface plasmon resonance. Biochemistry.

[11] Schaeffer P.M., Headlam M.J., Dixon N.E. (2005). Protein—Protein interactions in the eubacterial replisome. IUBMB Life.

[12] Mulugu S., Potnis A., Shamsuzzaman, Taylor J., Alexander K., Bastia D. (2001). Mechanism of termination of DNA replication of Escherichia coli involves helicase-contrahelicase interaction. Proc. Natl. Acad. Sci. USA.

[13] Pandey M., Elshenawy M.M., Jergic S., Takahashi M., Dixon N.E., Hamdan S.M., Patel S.S. (2015). Two mechanisms coordinate replication termination by the Escherichia coli Tus-Ter complex. Nucl. Acids Res..

[14] Berghuis B.A., Dulin D., Xu Z.Q., van Laar T., Cross B., Janissen R., Jergic S., Dixon N.E., Depken M., Dekker N.H. (2015). Strand separation establishes a sustained lock at the Tus-Ter replication fork barrier. Nat. Chem. Biol..

[15] Elshenawy M.M., Jergic S., Xu Z.Q., Sobhy M.A., Takahashi M., Oakley A.J., Dixon N.E., Hamdan S.M. (2015). Replisome speed determines the efficiency of the Tus-Ter replication termination barrier. Nature.

[16] Kamada K., Horiuchi T., Ohsumi K., Shimamoto N., Morikawa K. (1996). Structure of a replication-terminator protein complexed with DNA. Nature.

[17] Moreau M.J., Schaeffer P.M. (2013). Dissecting the salt dependence of the Tus-Ter protein-DNA complexes by high-throughput differential scanning fluorimetry of a GFP-tagged Tus. Mol. Biosyst..

[18] Coskun-Ari F.F., Hill T.M. (1997). Sequence-specific interactions in the Tus-Ter complex and the effect of base pair substitutions on arrest of DNA replication in Escherichia coli. J. Biol. Chem..

[19] Horiuchi T., Nishitani H., Kobayashi T. (1995). A new type of E. coli recombinational hotspot which requires for activity both DNA replication termination events and the Chi sequence. Adv. Biophys..

[20] Mohanty B.K., Bairwa N.K., Bastia D. (2009). Contrasting roles of checkpoint proteins as recombination modulators at Fob1-Ter complexes with or without fork arrest. Eukaryot. Cell.

[21] Rothstein R., Michel B., Gangloff S. (2000). Replication fork pausing and recombination or “gimme a break”. Genes Dev..

[22] Moolman M.C., Tiruvadi Krishnan S., Kerssemakers J.W., de Leeuw R., Lorent V., Sherratt D.J., Dekker N.H. (2016). The progression of replication forks at natural replication barriers in live bacteria. Nucl. Acids Res..

[23] Roecklein B., Pelletier A., Kuempel P. (1991). The tus gene of Escherichia coli: Autoregulation, analysis of flanking sequences and identification of a complementary system in *Salmonella typhimurium*. Res. Microbiol..

[24] Roecklein B.A., Kuempel P.L. (1992). In vivo characterization of tus gene expression in Escherichia coli. Mol. Microbiol..

[25] Enyeart P.J., Chirieleison S.M., Dao M.N., Perutka J., Quandt E.M., Yao J., Whitt J.T., Keatinge-Clay A.T., Lambowitz A.M., Ellington A.D. (2013). Generalized bacterial genome editing using mobile group II introns and Cre-lox. Mol. Syst. Biol..

[26] Bidnenko V., Ehrlich S.D., Michel B. (2002). Replication fork collapse at replication terminator sequences. EMBO J..

[27] Sharma B., Hill T.M. (1995). Insertion of inverted Ter sites into the terminus region of the Escherichia coli chromosome delays completion of DNA replication and disrupts the cell cycle. Mol. Microbiol..

[28] Bidnenko V., Lestini R., Michel B. (2006). The Escherichia coli UvrD helicase is essential for Tus removal during recombination-dependent replication restart from Ter sites. Mol. Microbiol..

[29] Esnault E., Valens M., Espeli O., Boccard F. (2007). Chromosome structuring limits genome plasticity in Escherichia coli. PLoS Genet..

[30] Poteete A.R. (2001). What makes the bacteriophage lambda Red system useful for genetic engineering: Molecular mechanism and biological function. FEMS Microbiol. Lett..

[31] Mei Q., Fitzgerald D.M., Liu J., Xia J., Pribis J.P., Zhai Y., Nehring R.B., Paiano J., Li H., Nussenzweig A. (2021). Two mechanisms of chromosome fragility at replication-termination sites in bacteria. Sci. Adv..

[32] Moreau M.J.J., Morin I., Askin S.P., Cooper A., Moreland N.J., Vasudevan S.G., Schaeffer P.M. (2012). Rapid determination of protein stability and ligand binding by differential scanning fluorimetry of GFP-tagged proteins. RSC Adv..

[33] Worning P., Jensen L.J., Hallin P.F., Staerfeldt H.H., Ussery D.W. (2006). Origin of replication in circular prokaryotic chromosomes. Environ. Microbiol..

[34] Touchon M., Rocha E.P. (2008). From GC skews to wavelets: A gentle guide to the analysis of compositional asymmetries in genomic data. Biochimie.

[35] Arakawa K., Tomita M. (2007). The GC skew index: A measure of genomic compositional asymmetry and the degree of replicational selection. Evol. Bioinform..

[36] Hendrickson H., Lawrence J.G. (2007). Mutational bias suggests that replication termination occurs near the dif site, not at Ter sites. Mol. Microbiol..

[37] Kono N., Arakawa K., Tomita M. (2012). Validation of bacterial replication termination models using simulation of genomic mutations. PLoS ONE.

[38] Kono N., Tomita M., Arakawa K. (2018). Accelerated Laboratory Evolution Reveals the Influence of Replication on the GC Skew in Escherichia coli. Genome Biol. Evol..

[39] Galli E., Ferat J.L., Desfontaines J.M., Val M.E., Skovgaard O., Barre F.X., Possoz C. (2019). Replication termination without a replication fork trap. Sci. Rep..

[40] Henderson T.A., Nilles A.F., Valjavec-Gratian M., Hill T.M. (2001). Site-directed mutagenesis and phylogenetic comparisons of the Escherichia coli Tus protein: DNA-protein interactions alone can not account for Tus activity. Mol. Genet. Genom..

[41] Willis N.A., Chandramouly G., Huang B., Kwok A., Follonier C., Deng C., Scully R. (2014). BRCA1 controls homologous recombination at Tus/Ter-stalled mammalian replication forks. Nature.

[42] Larsen N.B., Sass E., Suski C., Mankouri H.W., Hickson I.D. (2014). The Escherichia coli Tus-Ter replication fork barrier causes site-specific DNA replication perturbation in yeast. Nat. Commun..

[43] Morin I., Dixon N.E., Schaeffer P.M. (2010). Ultrasensitive detection of antibodies using a new Tus-Ter-lock immunoPCR system. Mol. Biosyst..

[44] Askin S.P., Schaeffer P.M. (2012). A universal immuno-PCR platform for comparative and ultrasensitive quantification of dual affinity-tagged proteins in complex matrices. Analyst.

[45] Natarajan S., Kaul S., Miron A., Bastia D. (1993). A 27 kd protein of E. coli promotes antitermination of replication in vitro at a sequence-specific replication terminus. Cell.

[46] Goodall D.J., Jameson K.H., Hawkins M., Rudolph C.J. (2021). A Fork Trap in the Chromosomal Termination Area Is Highly Conserved across All Escherichia coli Phylogenetic Groups. Int. J. Mol. Sci..

[47] Dahdah D.B., Morin I., Moreau M.J., Dixon N.E., Schaeffer P.M. (2009). Site-specific covalent attachment of DNA to proteins using a photoactivatable Tus-Ter complex. Chem. Commun..

[48] Moreau M.J., Morin I., Schaeffer P.M. (2010). Quantitative determination of protein stability and ligand binding using a green fluorescent protein reporter system. Mol. Biosyst..

[49] Morin I., Schaeffer P.M., Askin S.P., Dixon N.E. (2012). Combining RNA-DNA swapping and quantitative polymerase chain reaction for the detection of influenza A nucleoprotein. Anal. Biochem..

[50] Volkmer B., Heinemann M. (2011). Condition-dependent cell volume and concentration of Escherichia coli to facilitate data conversion for systems biology modeling. PLoS ONE.

[51] Meijerink J., Mandigers C., van de Locht L., Tonnissen E., Goodsaid F., Raemaekers J. (2001). A novel method to compensate for different amplification efficiencies between patient DNA samples in quantitative real-time PCR. J. Mol. Diagn..

[52] Isaacs F.J., Carr P.A., Wang H.H., Lajoie M.J., Sterling B., Kraal L., Tolonen A.C., Gianoulis T.A., Goodman D.B., Reppas N.B. (2011). Precise manipulation of chromosomes in vivo enables genome-wide codon replacement. Science.

[53] Edgar R.C. (2004). MUSCLE: A multiple sequence alignment method with reduced time and space complexity. BMC Bioinform..

[54] Stamatakis A. (2014). RAxML version 8: A tool for phylogenetic analysis and post-analysis of large phylogenies. Bioinformatics.

[55] Letunic I., Bork P. (2019). Interactive Tree Of Life (iTOL) v4: Recent updates and new developments. Nucleic Acids Res..

