# Supplementary material for: Delineation of the Ancestral Tus-Dependent Replication Fork Trap
Source: Int J Mol Sci. 2021 Dec 16;22(24):13533. doi: 10.3390/ijms222413533 (PMC8707476; doi:10.3390/ijms222413533)
Supplement: Supplementary file 1 [file ijms-22-13533-s001.zip › ijms-1488716-supplementary.pdf]

SUPPLEMENTARY DATA

Delineation of the ancestral Tus-dependent replication fork trap

Casey J. Toft[1,2], Morgane J. J. Moreau[1], Jiri Perutka[3], Savitri Mandapati[3], Peter Enyeart[3], Alanna E. Sorenson[1], Andrew D. Ellington[3] and Patrick M. Schaeffer[1,2*]

[1] Molecular and Cell Biology, College of Public Health, Medical and Veterinary Sciences, James Cook University, Douglas, QLD, 4811

[2] Centre of Tropical Bioinformatics and Molecular Biology, James Cook University, Douglas, QLD, 4811, Australia

[3] Institute for Cell and Molecular Biology, University of Texas at Austin, Austin, TX, 78712, USA

* To whom correspondence should be addressed. Tel: +61 (0) 7 4781 4448; Fax: +61 (0) 7 4781 6078; Email: patrick.schaeffer@jcu.edu.au

MATERIALS AND OTHER RESOURCES

REAGENT or RESOURCE	SOURCE	IDENTIFIER
Antibodies		
Chicken anti-GFP IgY	Abcam	ab92456
HRP-conjugated goat anti-IgY (Jackson 103-035-155)	Jackson ImmunoResearch Laboratories	103-035-155
Bacterial Strains		
E. coli KRX	Promega	Cat#: L3002
BL21(*DE3*)RIPL	Stratagene	Cat#: 230280
Dickeya paradisiaca (strain Ech703)	RefSeq	NC_012880
Edwardsiella tarda (strain EIB202)	RefSeq	NC_013508
Proteus mirabilis (strain HN2p)	RefSeq	NZ_CP046048
Xenorhabdus nematophila (strain ATCC 19061)	RefSeq	NC_014228
Salmonella enterica serovar *Typhimurium* (strain LT2)	RefSeq	NC_003197
Escherichia coli (strain K12 substr. MG1655)	RefSeq	U00096
Cedecea neteri (strain ND14a)	RefSeq	NZ_CP009459
Recombinant DNA		
Plasmid: pPMS1259	Schaeffer Lab	(Dahdah et al., 2009)

Chemicals, Peptides, and Recombinant Proteins		
His_6-Tus-GFP	Schaeffer Lab	N/A
SIGMAFAST™ 3,3′-Diaminobenzidine tablets	Sigma	d4418
SensiMix SYBR & fluorescein mastermix	Bioline	QT615-05
Critical Commercial Assays		
NEBNext Ultra DNA library preparation kit	New England BioLabs	E7370S
QuantiFluor® dsDNA System	Promega	E2670
Rapid Sequencing protocol (FLO-MIN106 R9 MinION)	Oxford Nanopore	SQK-RAD004
Deposited Data		
ChIP-Seq data set and KRX assembly	NCBI GEO	Accession: GSE163680
Oligonucleotides		
See Supplementary Data for full list of sequences and genomic loci for amplification of *oriC* and *Ter* regions by qPCR		
Software and Algorithms		
MinKNOW	Oxford Nanopore Technologies	https://github.com/nanoporetech/minknow_api

Trimmomatic	(Bolger et al., 2014)	http://www.usadellab.org/cms/?page=trimmomatic
Porechop	(Wick et al., 2017)	https://github.com/rrwick/Porechop
Flye	(Kolmogorov et al., 2019)	https://github.com/fenderglass/Flye
Racon	(Vaser et al., 2017)	https://github.com/isovic/racon
Pilon	(Walker et al., 2014)	https://github.com/broadinstitute/pilon
Quast	(Gurevich et al., 2013)	http://quast.sourceforge.net/quast
Prokka	(Seemann, 2014)	https://github.com/tseemann/prokka
Bowtie2	(Langmead and Salzberg, 2012)	http://bowtie-bio.sourceforge.net/bowtie2/index.shtml
Samtools	(Li et al., 2009)	http://www.htslib.org/
Circleator	(Crabtree et al., 2014)	http://jonathancrabtree.github.io/Circleator/

genomeCoverageBed	(Quinlan and Hall, 2010)	https://bedtools.readthedocs.io/en/latest/content/tools/genomecov.html
blastn	(Altschul et al., 1990)	https://ftp.ncbi.nlm.nih.gov/blast/executables/blast+/LATEST/
Interactive Genomics Viewer (IGV)	(Thorvaldsdottir et al., 2013)	http://software.broadinstitute.org/software/igv/
EzMol Molecular display wizard	(Reynolds et al., 2018)	http://www.sbg.bio.ic.ac.uk/ezmol/
InterPro Protein Data Bank	(Mitchell et al., 2019)	https://www.ebi.ac.uk/interpro/
iTol	(Letunic and Bork, 2019)	https://itol.embl.de/
RAxML	(Stamatakis, 2014)	https://cme.h-its.org/exelixis/web/software/raxml/
MUSCLE	(Edgar, 2004)	http://www.drive5.com/muscle/downloads.htm
ImageJ	(Schneider et al., 2012)	https://imagej.nih.gov/ij/index.html

GraphPad 8	GraphPad Software	https://www.graphpad.com/scientific-software/prism/

CONTACT FOR REAGENT AND RESOURCE SHARING

Further information and requests for resources and reagents should be directed to the corresponding author: Patrick.schaeffer@jcu.edu.au.

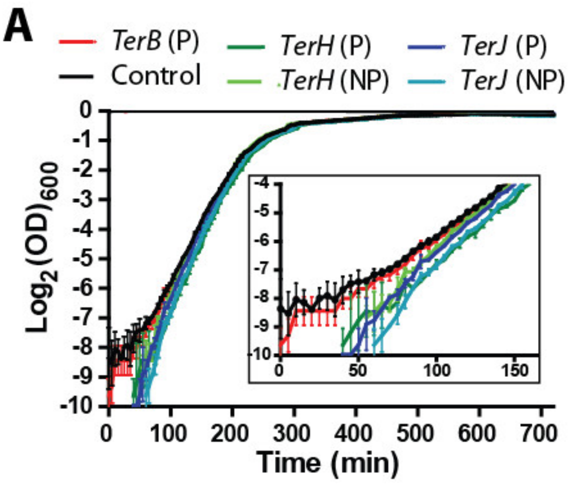

Figure S1: Effect of ectopic Ter sites on the growth rate of E. coli BL21(DE3). *TerB*, *TerH* and *TerJ* were inserted ~ 930 kpb downstream of *oriC* in the permissive (P) or non-permissive (NP) orientation. (A) Growth rates were measured in independent triplicates. Error bars represent SD. A culture of wild type BL21(DE3) was grown as a control. Growth rates were determined from the slopes of the linear regressions performed between 100 and 210 minutes (see Table 1 in the main text). Doubling time (T_D) was calculated as 1/growth rate (n=3, except for TerH (NP), n=2). Reproduced with permission from Moreau, PhD thesis, James Cook University (2013). Thesis can be downloaded using the following link: ***https://researchonline.jcu.edu.au/31903/1/31903_Moreau_2013_thesis.pdf***).

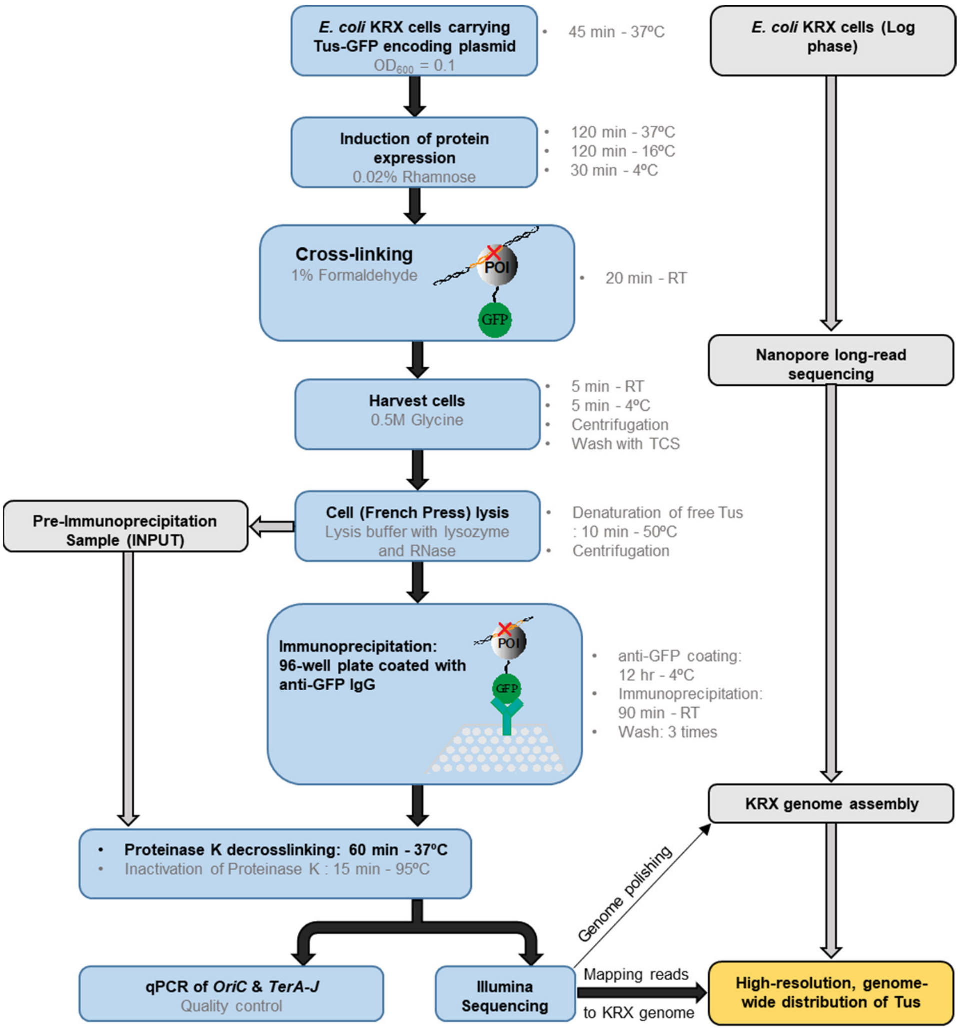

Figure S2: ChIP-qPCR and ChIP-Seq process using a 96-well plate format coated with anti-GFP IgG, and genome assembly for *E. coli* KRX strain. See Star Methods section for detailed procedures.

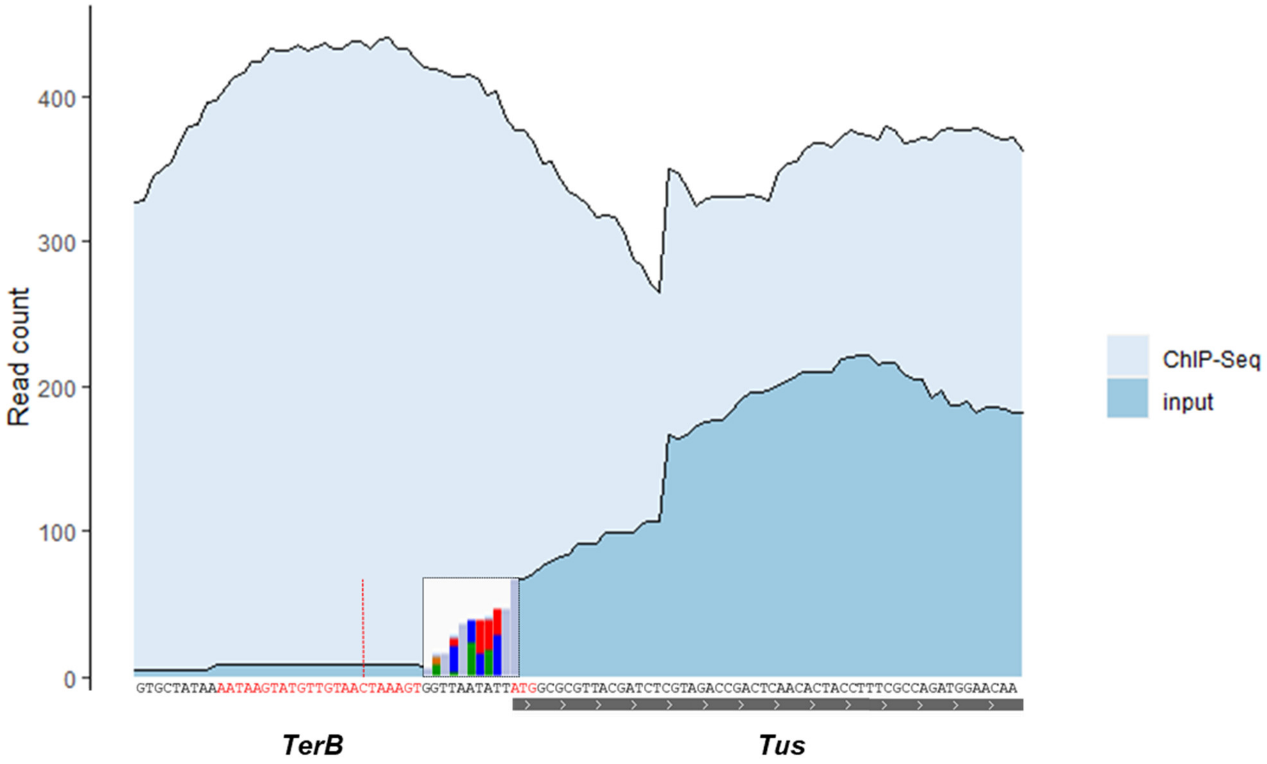

Figure S3: Nucleotide read count at genomic *TerB* and *tus* gene loci for immunoprecipitated DNA (ChIP-Seq) and non-immunoprecititated DNA (Input). The boxed bar chart between *TerB* and *tus* shows an ambiguous 10 nucleotide sequence with partial identity between the plasmid and genome sequences upstream the start codon (ATG) of the *tus* sequence. The data show that the high read count originating from the plasmid *tus* sequence (i.e. misaligned to the genomic *tus* locus) does not bias the read count at *TerB*. The dashed red line indicates the C(6) position in *TerB* critical for the formation of the Tus-*Ter*-lock structure.

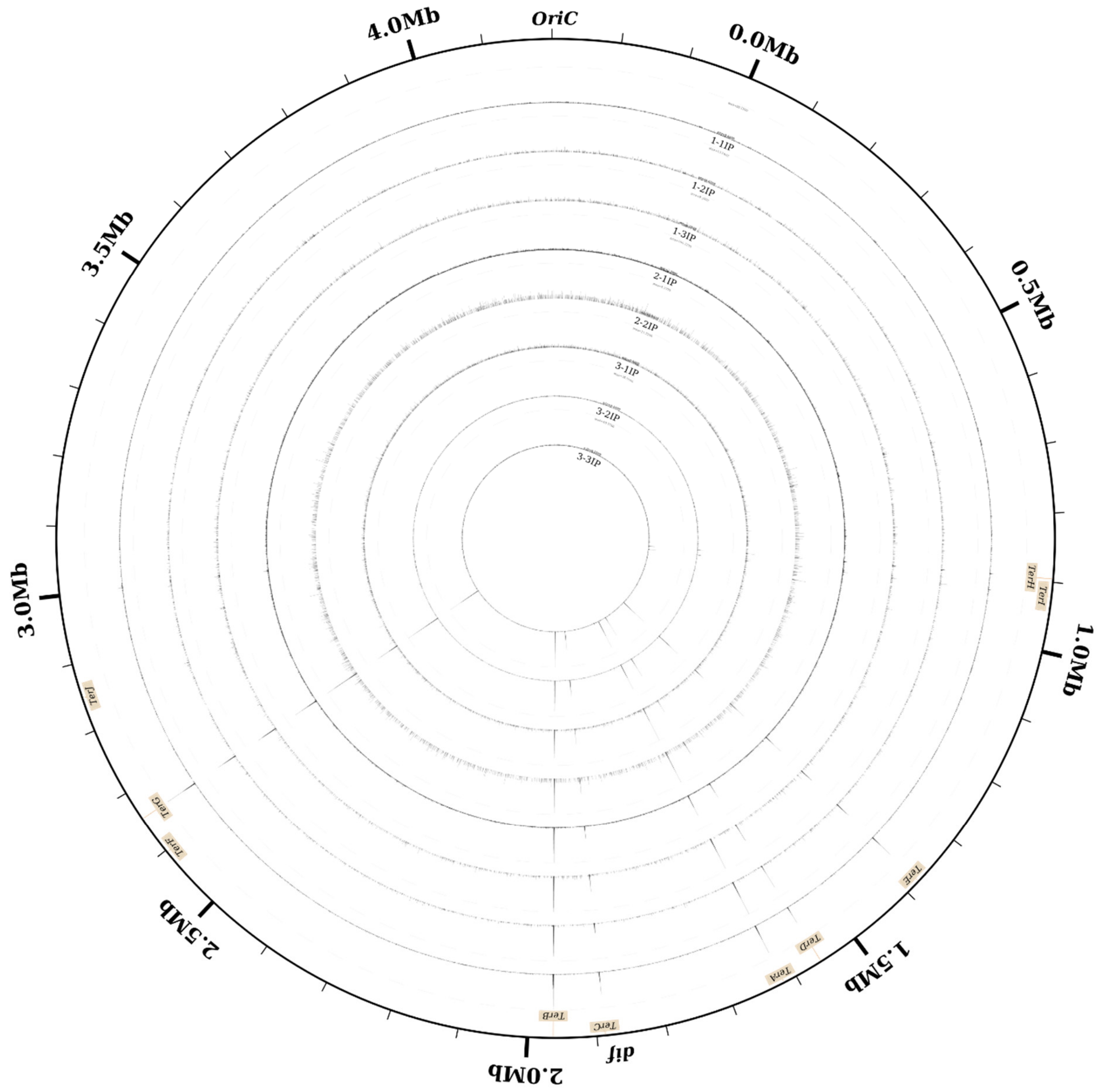

Figure S4: Individually mapped ChIP-Seq coverage for experimental and biological replicates (n = 8). Three biological replicates of immunoprecipitated DNA (ChIP) are shown each consisting of 2-3 technical replicates as indicated. The difference in peak height between different *Ter* sites is consistent despite varying depths of sequencing between replicates. See Figure 2 for pooled data.

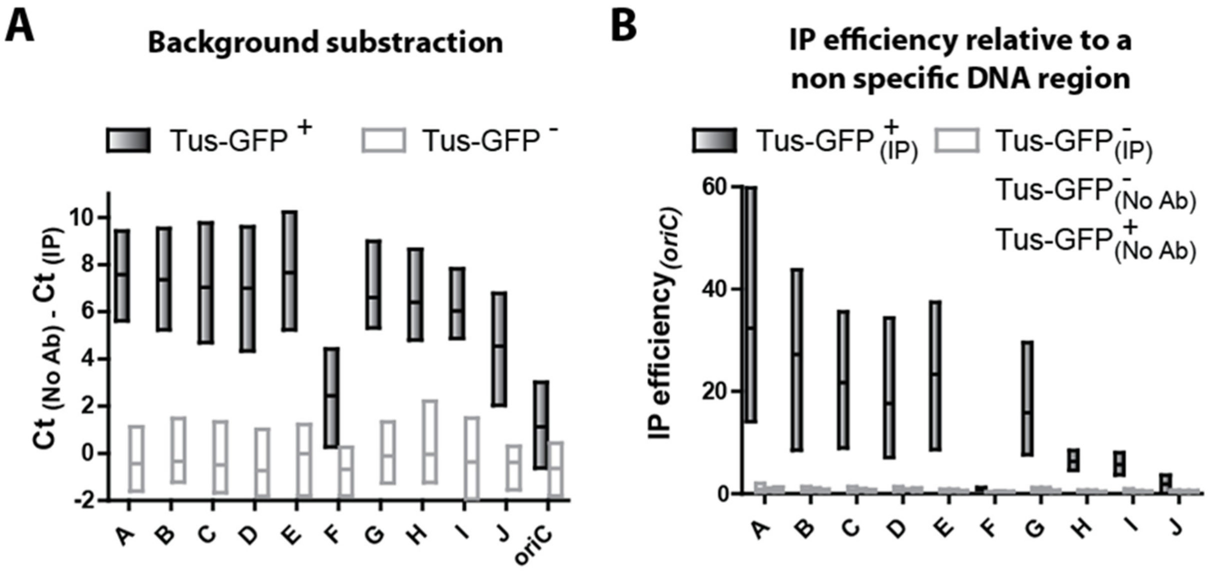

Figure S5: Distribution of Tus-GFP on *Ter* sites in *E. coli* KRX cells by ChIP-qPCR. (A) Difference in Ct-values between immunoprecipitated DNA (IP) and background control experiments in absence of anti-GFP (No Ab) obtained for Tus-GFP$^+$ and Tus-GFP$^-$ control KRX cells. (B) IP efficiency of *Ter* sites relative to a non-specific *oriC* region obtained for Tus-GFP$^+$ and Tus-GFP$^-$ control KRXcells in the presence (IP) or absence of anti-GFP IgG antibody (No Ab). Floating bars represent minimum, maximum and mean values. Reproduced with permission from Moreau, PhD thesis, James Cook University (2013). Thesis can be downloaded at: https://researchonline.jcu.edu.au/31903/1/31903_Moreau_2013_thesis.pdf.

A

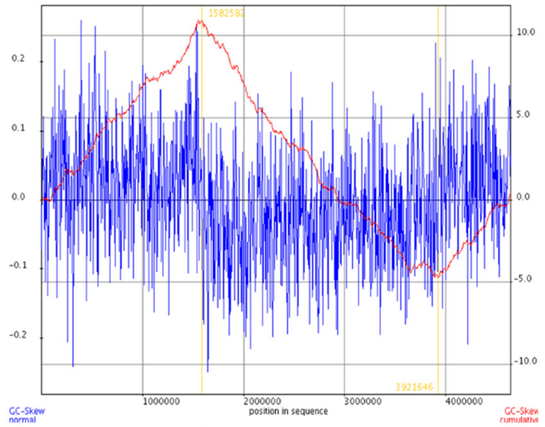

1,582,582

B

	TerA	*TerB*	*TerC*	*dif*	GC skew transition midpoint
Location in *E. coli* genome (bp)	1,341,745	1,684,226	1,609,157	1,590,777	1,582,582
In vivo replication arrest (%)	0.19	0.14	0.85	-	-
Fraction of arrest Hypothesis 1	0.161	0.119	0.72	-	-
Fraction of arrest Hypothesis 2	0.183	-	0.817	-	-
Fraction of arrest Hypothesis 3	-	-	-	1	-
					**Distance from GC skew (bp)**
**Predicted GC-inflection Hypothesis 1 (bp)**		1,575,037			-7545
**Predicted GC-inflection Hypothesis 2 (bp)**		1,560,221			-22361
**Predicted GC-inflection Hypothesis 3**		1,590,777			8195

Figure S6: (A) GC skew transition midpoint calculated with a 1000 bp sliding window for *E. coli* MG1655 used in the *in vivo* replication arrest study by Duggin and Bell, (2009). (B) Hypothetical GC skew transition midpoint loci using various fork arrest scenarios based on the ensemble and fractional distribution of replication fork collision loci at functional *Ter* sites. Only *Ter* sites with significant replication fork arrest activity (*TerA*, *TerB* and *TerC*) are included. *Dif* site is also shown for comparison. Locations of *TerA-C* and *dif* in *E. coli* MG1655 are indicated in the table.

Hypothesis 1: Replication fork arrest occurs with the fractional distribution of Y forks reported by Duggin and Bell, 2009.

Hypothesis 2: Replication fork arrest occurs at *TerA* and *TerC* with equal fractional distribution i.e. fork arrest only occurs at *TerA* and *TerC* and are never breached.

Hypothesis 3: Replication fork arrest occurs at *dif* site

Conclusion: the terminal GC-skew swith derived from hypothesis 1 (i.e. 1,575,037 bp) involving *TerA-C* deviates the least from the switch point derived from a sliding 1,000 bp cumulative GC-skew (i.e. 1,582,582 bp) by only 7.5 kbp. It is important to note that the *dif* site is located 8 kbp from the terminal GC-skew switch point on the other chromosomal arm.

A

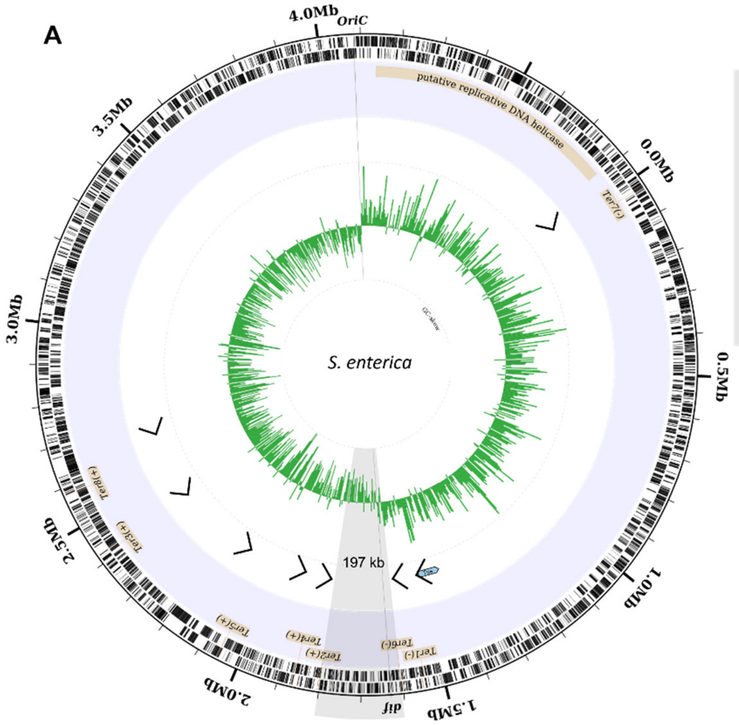

	NP	lock		P	tus
Ter1	ATATA	G	TATGTTGTAACT	AAAGT	GAGTGATACCATG
Ter2	--TA-	-	------------	----C	
Ter3	C-T--	-	------------	---TA	
Ter4	TATA-	-	------------	---TA	
Ter5	GATG-	-	------------	--T-A	
Ter6	TA-CT	-	------------	---TA	
Ter7	T-GCT	-	------------	--GA-	
pTer8	GCGG-	-	--A---------	--T-C	
TerB	-ATA-	-	------------	-----	

B

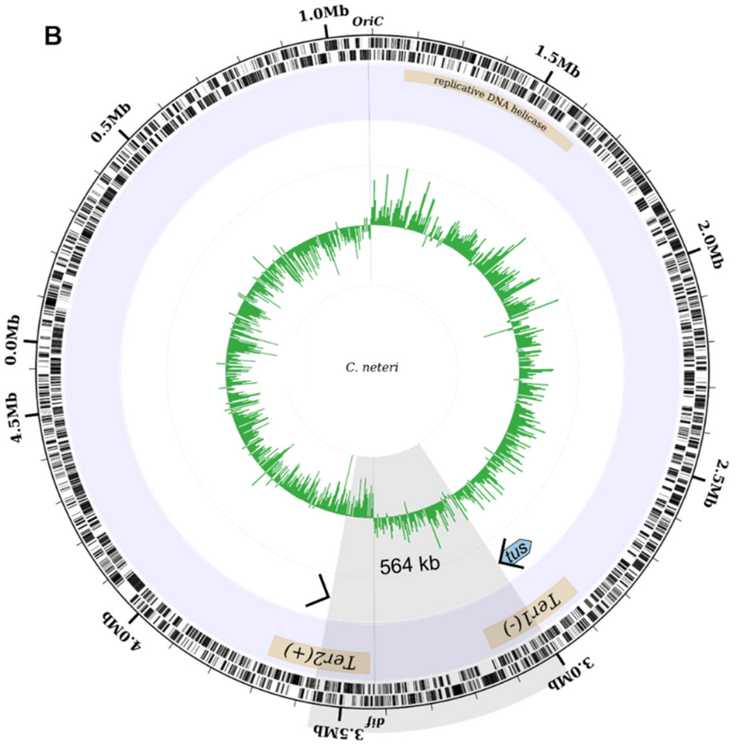

	NP	lock		P	tus
Ter1	TATTA	G	TATGTTGTAACT	AATGT	GAGTTACTATG
Ter2	-T---	-	------------	--AAC	
TerB	A--A-	-	------------	--A--	

C

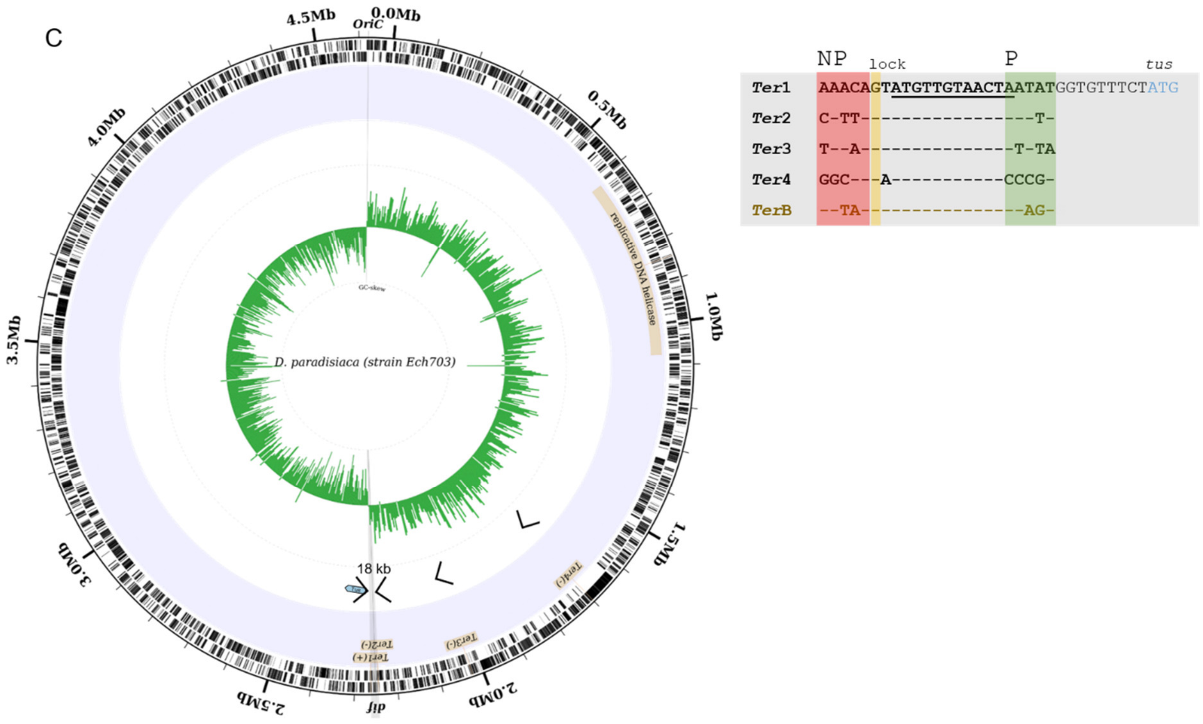
4.5Mb
0.0Mb
OriC
0.5Mb
1.0Mb
1.5Mb
2.0Mb
2.5Mb
3.0Mb
3.5Mb
4.0Mb
replicative DNA helicase
GC-skew
D. paradisiaca (strain Ech703)
18 kb
Ter4(-)
Ter3(-)
Ter2(-)
Ter1(+)
dif
NP
lock
P
tus
Ter1 AAACAGTATGTTGTAACTAATATGGTGTTTCTATG
Ter2 C-TT-----------------T-
Ter3 T--A---------------T-TA
Ter4 GGC---A-----------CCCG-
TerB --TA-----------------AG-

D

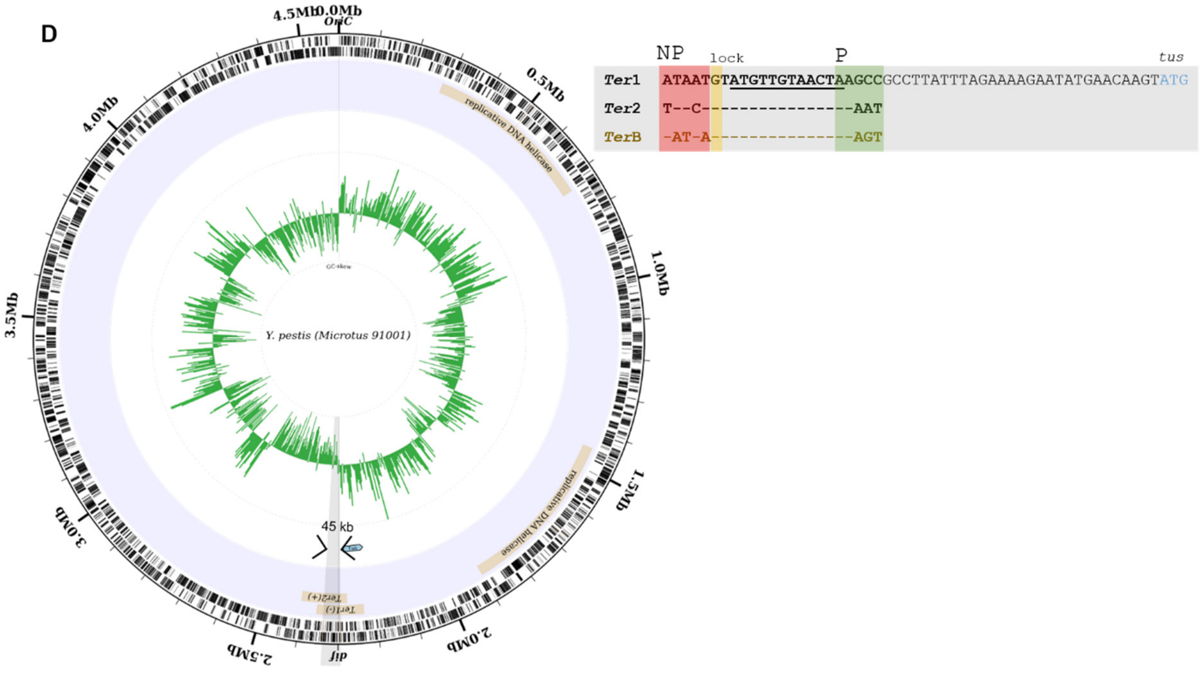
4.5Mb
0.0Mb
OriC
0.5Mb
1.0Mb
1.5Mb
2.0Mb
2.5Mb
3.0Mb
3.5Mb
4.0Mb
replicative DNA helicase
replicative DNA helicase
GC-skew
Y. pestis (Microtus 91001)
45 kb
Ter2(+)
Ter1(-)
dif
NP
lock
P
tus
Ter1 ATAATGTATGTTGTAACTAAGCCGCCTTATTTAGAAAAGAATATGAACAAGTATG
Ter2 T--C--------------AAT
TerB -AT-A-------------AGT

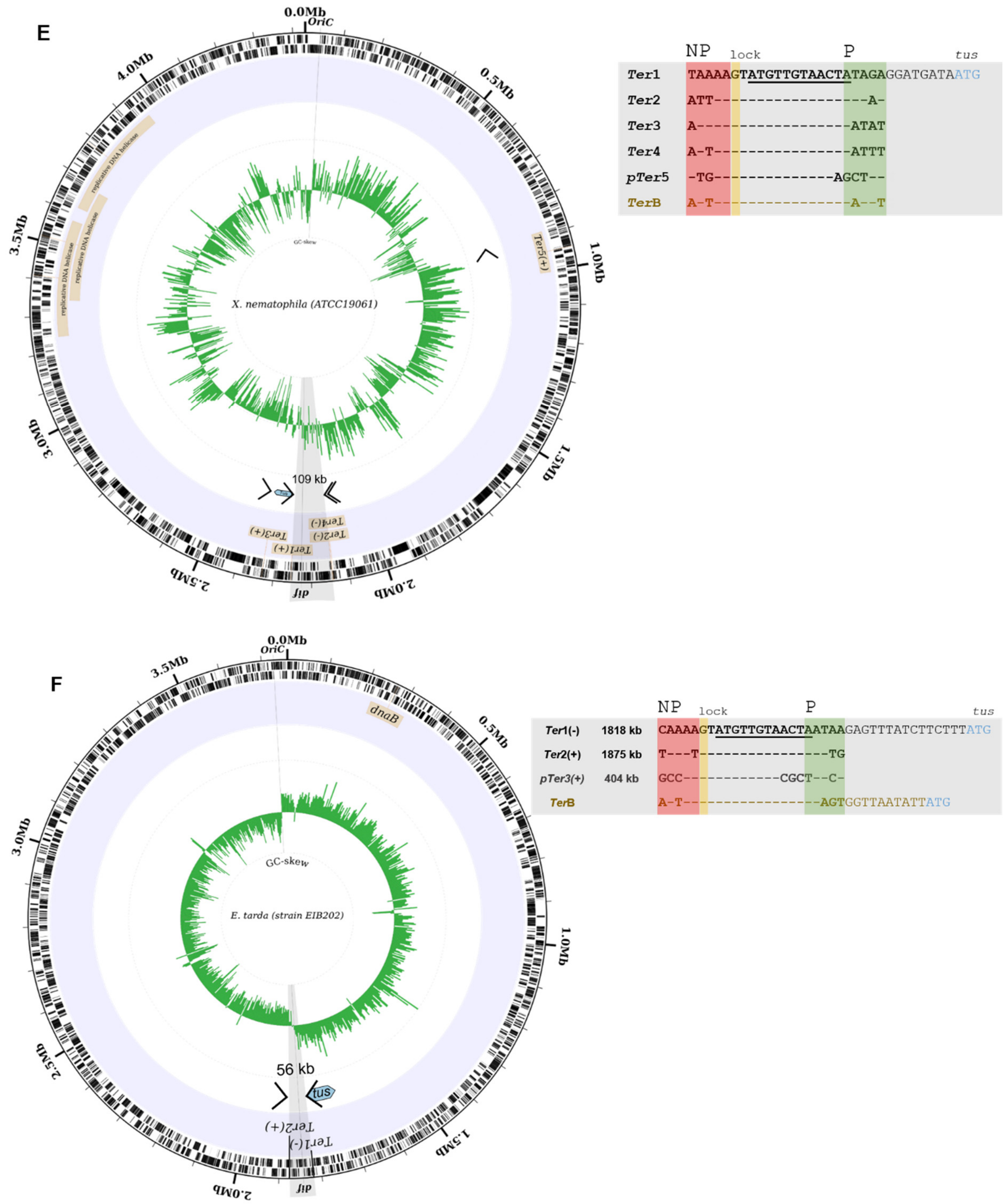

Figure S7: Circular representation of (A) *Salmonella enterica* serovar Typhimurium LT2 ***T, (B) Cedecae netari, (C) Dickeya paradisiaca, (D) Yersinia pestis (E) Xenorhabdus nematophila*** **and** ***(F) Edwardsiella tarda*** **chromosomes and their** ***Ter*** **sequences.** From the outside of the circle: Forward and reverse genes; genomic locations of identified *Ter* sites involved in DNA replication termination; and GC-skew with a 5000 bp sliding window. In each alignment, *Ter1* represents the *Ter* sequence adjacent to *tus* in the indicated chromosome followed by the RBS and start codon of *tus*. The G(6) complementary to C(6) is highlighted in yellow and the strictly conserved 12

bp core sequence is underlined. The non-permissive face (NP) is highlighted in red and the permissive face (P) is highlighted in green.

Table S1: Chromosomal fork trap architecture and classification for selected bacteria.

Family	Species	# of *Ter* sites	Fork trap size (kb)	Vicinal *Ter* sequence	Vicinal *Ter* identity	Identity to *E .coli* Tus (%)	Fork-trap classification	
Enterobacteriaceae	*Escherichia coli*	6*	267	AATAAGTATGTTGTAACTAAAGT	-	-	Type II	
	Salmonella enterica	8	197	ATATAGTATGTTGTAACTAAAGT	20/23	80	Type II	
	Cronobacter dublinensis	9	47	ATAAAGTATGTTGTAACTAATGT	20/23	65	Type II	
	Atlantibacter hermannii	8	43	AAATAGTATGTTGTAACTAAAGG	20/23	61.8	Type II	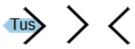
	Shimwellia blattae	5	72	AATAAGCATGTTGTAACTAAAGA	21/23	60	Type II	
	Buttiauxella agrestis	5	297	CTTTAGTATGTTGTAACTAATGG	18/23	60.5	Type II	
	Cedecea netari str. FDAARGOS	3	507	CATTAGTATGTTGTAACTAAAGT	21/23	59.2	Type I	
	Cedecea neteri str. ND14a	2	564	TATTAGTATGTTGTAACTAATGT	20/23	58.6	Type I	
Erwiniaceae	*Pantoea agglomerans*	4	246	TTATAGTATGTTGTAACTATAAA	16/23	55.4	Type I	
Pectobacteriaceae	*Sodalis praecaptivus*	2	111	GTATAGTATGTTGTAACTAATAG	16/23	50.2	Type I	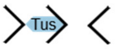
	Dickeya Paradisiaca	4	18	AAACAGTATGTTGTAACTAATAT	19/23	53.9	Type I	
Hafniaceae	*Edwardsiella tarda*	2	58	CAAAAGTATGTTGTAACTAATAA	18/23	48.3	Type I	
Yersiniaceae	*Yersinia pestis*	2	45	ATAATGTATGTTGTAACTAAGCC	17/23	52.7	Type I	
Morganellaceae	*Xenorhabdus nematophila*	4	109	TAAAAGTATGTTGTAACTATAGA	19/23	46.3	Type I	
	Proteus mirabilis	2	137	TAATTGTATGTTGTAACTAAATA	17/23	50.8	Type I	

Fork trap size corresponds to the distance between the two innermost Ter sites of opposite polarity expressed in kb. Underlined bases represent a continuous identical sequence shared between all Ter sequences vicinal to tus starting at the G(6).

REFERENCES

Altschul SF, Gish W, Miller W, Myers EW, Lipman DJ. Basic local alignment search tool. J Mol Biol 1990;215(3):403-10.

Bolger AM, Lohse M, Usadel B. Trimmomatic: a flexible trimmer for Illumina sequence data. Bioinformatics 2014;30(15):2114-20.

Crabtree J, Agrawal S, Mahurkar A, Myers GS, Rasko DA, White O. Circleator: flexible circular visualization of genome-associated data with BioPerl and SVG. Bioinformatics 2014;30(21):3125-7.

Dahdah DB, Morin I, Moreau MJ, Dixon NE, Schaeffer PM. Site-specific covalent attachment of DNA to proteins using a photoactivatable Tus-Ter complex. Chem Commun (Camb) 2009(21):3050-2.

Edgar RC. MUSCLE: a multiple sequence alignment method with reduced time and space complexity. BMC Bioinformatics 2004;5:113.

Gurevich A, Saveliev V, Vyahhi N, Tesler G. QUAST: quality assessment tool for genome assemblies. Bioinformatics 2013;29(8):1072-5.

Kolmogorov M, Yuan J, Lin Y, Pevzner PA. Assembly of long, error-prone reads using repeat graphs. Nat Biotechnol 2019;37(5):540-546.

Langmead BSalzberg SL. Fast gapped-read alignment with Bowtie 2. Nat Methods 2012;9(4):357-9.

Letunic IBork P. Interactive Tree Of Life (iTOL) v4: recent updates and new developments. Nucleic Acids Res 2019;47(W1):W256-W259.

Li H, Handsaker B, Wysoker A, Fennell T, Ruan J, Homer N, Marth G, Abecasis G, Durbin R, Genome Project Data Processing S. The Sequence Alignment/Map format and SAMtools. Bioinformatics 2009;25(16):2078-9.

Mitchell AL, Attwood TK, Babbitt PC, Blum M, Bork P, Bridge A, Brown SD, Chang HY, El-Gebali S, Fraser MI, Gough J, Haft DR, Huang H, Letunic I, Lopez R, Luciani A, Madeira F, Marchler-Bauer A, Mi H, Natale DA, Necci M, Nuka G, Orengo C, Pandurangan AP, Paysan-Lafosse T, Pesseat S, Potter SC, Qureshi MA, Rawlings ND, Redaschi N, Richardson LJ, Rivoire C, Salazar GA, Sangrador-Vegas A, Sigrist CJA, Sillitoe I, Sutton GG, Thanki N, Thomas PD, Tosatto SCE, Yong SY, Finn RD. InterPro in 2019: improving coverage, classification and access to protein sequence annotations. Nucleic Acids Res 2019;47(D1):D351-D360.

Quinlan ARHall IM. BEDTools: a flexible suite of utilities for comparing genomic features. Bioinformatics 2010;26(6):841-2.

Reynolds CR, Islam SA, Sternberg MJE. EzMol: A Web Server Wizard for the Rapid Visualization and Image Production of Protein and Nucleic Acid Structures. J Mol Biol 2018;430(15):2244-2248.

Schneider CA, Rasband WS, Eliceiri KW. NIH Image to ImageJ: 25 years of image analysis. Nat Methods 2012;9(7):671-5.

Seemann T. Prokka: rapid prokaryotic genome annotation. Bioinformatics 2014;30(14):2068-9.

Stamatakis A. RAxML version 8: a tool for phylogenetic analysis and post-analysis of large phylogenies. Bioinformatics 2014;30(9):1312-3.

Thorvaldsdottir H, Robinson JT, Mesirov JP. Integrative Genomics Viewer (IGV): high-performance genomics data visualization and exploration. Brief Bioinform 2013;14(2):178-92.

Vaser R, Sovic I, Nagarajan N, Sikic M. Fast and accurate de novo genome assembly from long uncorrected reads. Genome Res 2017;27(5):737-746.

Walker BJ, Abeel T, Shea T, Priest M, Abouelliel A, Sakthikumar S, Cuomo CA, Zeng Q, Wortman J, Young SK, Earl AM. Pilon: an integrated tool for comprehensive microbial variant detection and genome assembly improvement. PLoS One 2014;9(11):e112963.

Wick RR, Judd LM, Gorrie CL, Holt KE. Completing bacterial genome assemblies with multiplex MinION sequencing. Microb Genom 2017;3(10):e000132.

ADDITIONAL RESOURCES

*This section provides the genomic loci of *Ter* sites in the KRX genome and orientation*

```
BLASTN 2.10.1+: Matrix: blastn matrix 1 -3,
Gap Penalties: Existence: 5, Extension: 2

Database: final_high_quality_krx_assembly.fasta
           1 sequences; 4,491,350 total letters
```

Query= TerAF

```
Length=24

>contig_2_pilon
Length=4491350

 Score = 48.1 bits (24),  Expect = 2e-07
 Identities = 24/24 (100%), Gaps = 0/24 (0%)
 Strand=Plus/Plus

Query  1        CAACCATTAAACCGATTCGCGGTC  24
                ||||||||||||||||||||||||
Sbjct  1619469  CAACCATTAAACCGATTCGCGGTC  1619492
```

Query= TerAR

```
Length=20

>contig_2_pilon
Length=4491350

 Score = 40.1 bits (20),  Expect = 3e-05
 Identities = 20/20 (100%), Gaps = 0/20 (0%)
 Strand=Plus/Minus

Query  1        AGTTGCGATTTCTCCCCTGG  20
                ||||||||||||||||||||
Sbjct  1619613  AGTTGCGATTTCTCCCCTGG  1619594
```

Query= TerBF

```
Length=22

>contig_2_pilon
Length=4491350

 Score = 44.1 bits (22),  Expect = 2e-06
 Identities = 22/22 (100%), Gaps = 0/22 (0%)
 Strand=Plus/Plus


Query  1        TTACCTCTGCCTGACACTACGC  22
                ||||||||||||||||||||||
Sbjct  1962934  TTACCTCTGCCTGACACTACGC  1962955
```

Query= TerBR

```
Length=23

>contig_2_pilon
Length=4491350
 Score = 46.1 bits (23),  Expect = 6e-07
 Identities = 23/23 (100%), Gaps = 0/23 (0%)
 Strand=Plus/Minus

Query  1        TGTTGAGTCGGTCTACGAGATCG  23
                |||||||||||||||||||||||
Sbjct  1963056  TGTTGAGTCGGTCTACGAGATCG  1963034
```

Query= TerCF

```
>contig_2_pilon
Length=4491350

 Score = 46.1 bits (23),  Expect = 6e-07
 Identities = 23/23 (100%), Gaps = 0/23 (0%)
 Strand=Plus/Plus

Query  1        CTGCATGTGGCACCTGTTAATGA  23
                |||||||||||||||||||||||
Sbjct  1887884  CTGCATGTGGCACCTGTTAATGA  1887906
```

Query= TerCR

```
Length=20

>contig_2_pilon
Length=4491350

 Score = 40.1 bits (20),  Expect = 3e-05
 Identities = 20/20 (100%), Gaps = 0/20 (0%)
 Strand=Plus/Minus

Query  1        GCTGTACGTCCGTTGTGCTA  20
                ||||||||||||||||||||
Sbjct  1888006  GCTGTACGTCCGTTGTGCTA  1887987
```

Query= TerDF

```
Length=25

>contig_2_pilon
Length=4491350

 Score = 50.1 bits (25),  Expect = 5e-08
 Identities = 25/25 (100%), Gaps = 0/25 (0%)
 Strand=Plus/Plus

Query  1        GGCATGATGTCGCGCttttttttATG  25
                |||||||||||||||||||||||||
Sbjct  1558486  GGCATGATGTCGCGCTTTTTTTTATG  1558510
```

Query= TerDR

```
Length=25

>contig_2_pilon
Length=4491350

 Score = 50.1 bits (25),  Expect = 5e-08
 Identities = 25/25 (100%), Gaps = 0/25 (0%)
 Strand=Plus/Minus

Query  1        GGGTATTAAGGAGTATTCCCCATGG  25
                |||||||||||||||||||||||||
Sbjct  1558610  GGGTATTAAGGAGTATTCCCCATGG  1558586
```

Query= TerEF

```
Length=20

>contig_2_pilon
```

```
Length=4491350

 Score = 40.1 bits (20),  Expect = 3e-05
 Identities = 20/20 (100%), Gaps = 0/20 (0%)
 Strand=Plus/Plus

Query  1        GAAGTCGCCGTCTGGTTTAT  20
                ||||||||||||||||||||
Sbjct  1377410  GAAGTCGCCGTCTGGTTTAT  1377429
```

Query= TerER

```
Length=20

>contig_2_pilon
Length=4491350

 Score = 40.1 bits (20),  Expect = 3e-05
 Identities = 20/20 (100%), Gaps = 0/20 (0%)
 Strand=Plus/Minus

Query  1        TACGGCGGAAGTTAATGGTC  20
                ||||||||||||||||||||
Sbjct  1377581  TACGGCGGAAGTTAATGGTC  1377562
```

Query= TerFF

```
Length=21

>contig_2_pilon
Length=4491350

 Score = 42.1 bits (21),  Expect = 8e-06
 Identities = 21/21 (100%), Gaps = 0/21 (0%)
 Strand=Plus/Plus

Query  1        CACATCTTCGGGAGTCGGTTC  21
                |||||||||||||||||||||
Sbjct  2596624  CACATCTTCGGGAGTCGGTTC  2596644
```

Query= TerFR

```
Length=22

>contig_2_pilon
Length=4491350

 Score = 44.1 bits (22),  Expect = 2e-06
 Identities = 22/22 (100%), Gaps = 0/22 (0%)
 Strand=Plus/Minus

Query  1        GGTTGAGTGGTAAACGCTGCTG  22
                ||||||||||||||||||||||
Sbjct  2596754  GGTTGAGTGGTAAACGCTGCTG  2596733
```

Query= TerGF

```
Length=20

>contig_2_pilon
Length=4491350

 Score = 40.1 bits (20),  Expect = 3e-05
 Identities = 20/20 (100%), Gaps = 0/20 (0%)
```

```
 Strand=Plus/Plus

Query  1        CCAAGCGAGTACCCCACCAG  20
                ||||||||||||||||||||
Sbjct  2656294  CCAAGCGAGTACCCCACCAG  2656313
```

Query= TerGR

```
Length=23

>contig_2_pilon
Length=4491350

 Score = 46.1 bits (23),  Expect = 6e-07
 Identities = 23/23 (100%), Gaps = 0/23 (0%)
 Strand=Plus/Minus

Query  1        CACGGTTGTATGTTGATCTCCCA  23
                |||||||||||||||||||||||
Sbjct  2656435  CACGGTTGTATGTTGATCTCCCA  2656413
```

Query= TerHF

```
Length=24

>contig_2_pilon
Length=4491350

 Score = 48.1 bits (24),  Expect = 2e-07
 Identities = 24/24 (100%), Gaps = 0/24 (0%)
 Strand=Plus/Plus

Query  1       TGAAGGACAAACTGGAAACGCTGA  24
               ||||||||||||||||||||||||
Sbjct  895054  TGAAGGACAAACTGGAAACGCTGA  895077
```

Query= TerHR

```
Length=20

>contig_2_pilon
Length=4491350

 Score = 40.1 bits (20),  Expect = 3e-05
 Identities = 20/20 (100%), Gaps = 0/20 (0%)
 Strand=Plus/Minus

Query  1       CAGACTACCGCCACCACAAT  20
               ||||||||||||||||||||
Sbjct  895201  CAGACTACCGCCACCACAAT  895182
```

Query= TerIF

```
Length=22

>contig_2_pilon
Length=4491350

 Score = 44.1 bits (22),  Expect = 2e-06
 Identities = 22/22 (100%), Gaps = 0/22 (0%)
 Strand=Plus/Plus

Query  1       ATTGCTGGAACGGTTGATTGCG  22
               ||||||||||||||||||||||
Sbjct  920804  ATTGCTGGAACGGTTGATTGCG  920825
```

Query= TerIR

```
Length=20
>contig_2_pilon
Length=4491350

 Score = 40.1 bits (20),  Expect = 3e-05
 Identities = 20/20 (100%), Gaps = 0/20 (0%)
 Strand=Plus/Minus

Query  1       CTCGCCGTCTTTACGTAGCA  20
               ||||||||||||||||||||
Sbjct  920921  CTCGCCGTCTTTACGTAGCA  920902
```

Query= TerJF

```
Length=20

>contig_2_pilon
Length=4491350

 Score = 40.1 bits (20),  Expect = 3e-05
 Identities = 20/20 (100%), Gaps = 0/20 (0%)
 Strand=Plus/Plus

Query  1        GACGATACGACGCACCGATG  20
                ||||||||||||||||||||
Sbjct  2849492  GACGATACGACGCACCGATG  2849511
```

Query= TerJR

```
Length=22
>contig_2_pilon
Length=4491350

 Score = 44.1 bits (22),  Expect = 2e-06
 Identities = 22/22 (100%), Gaps = 0/22 (0%)
 Strand=Plus/Minus

Query  1        CTGGTGATGCCGAACATGGAAG  22
                ||||||||||||||||||||||
Sbjct  2849641  CTGGTGATGCCGAACATGGAAG  2849620
```

Query= OriCF

```
Length=22
>contig_2_pilon
Length=4491350

 Score = 44.1 bits (22),  Expect = 2e-06
 Identities = 22/22 (100%), Gaps = 0/22 (0%)
 Strand=Plus/Plus

Query  1        CGCACTGCCCTGTGGATAACAA  22
                ||||||||||||||||||||||
Sbjct  4205199  CGCACTGCCCTGTGGATAACAA  4205220
```

Query= OriCR

```
Length=22
>contig_2_pilon
Length=4491350

 Score = 44.1 bits (22),  Expect = 2e-06
 Identities = 22/22 (100%), Gaps = 0/22 (0%)
 Strand=Plus/Minus

Query  1        CCCTCATTCTGATCCCAGCTTA  22
                ||||||||||||||||||||||
Sbjct  4205313  CCCTCATTCTGATCCCAGCTTA  4205292
```
